# Gaudichaudione H Inhibits Inflammatory Responses in Macrophages and Dextran Sodium Sulfate-Induced Colitis in Mice

**DOI:** 10.3389/fphar.2019.01561

**Published:** 2020-01-17

**Authors:** Yiwen Jiang, Lianbo Xiao, Wenwei Fu, Yuexun Tang, Peeraphong Lertnimitphun, Nami Kim, Changwu Zheng, Hongsheng Tan, Yue Lu, Hongxi Xu

**Affiliations:** ^1^ School of Pharmacy, Shanghai University of Traditional Chinese Medicine, Shanghai, China; ^2^ Institute of Arthritis Research, Shanghai Academy of Chinese Medical Sciences, Guanghua Integrative Medicine Hospital, Shanghai, China

**Keywords:** gaudichaudione H, macrophages, colitis, nitric oxide, nuclear factor-κB, mitogen-activated protein kinases

## Abstract

Macrophages-involved inflammation is considered to induce the damage in various diseases. Herein, novel therapeutics inhibiting over-activation of macrophages could prove an effective strategy to prevent inflammation-related diseases. Gaudichaudione H (GH), which is a natural small molecular compound isolated from *Garcinia oligantha* Merr. (Clusiaceae) has previously been demonstrated its anti-cancer effects on several cancer cell lines. However, no report has been published about the anti-inflammatory effect of GH to date. This study aims to examine the anti-inflammatory effects and potential molecular mechanism of GH, and provide new insights toward the treatment of inflammation. GH inhibited nitric oxide (NO) production, inducible nitric oxide synthase (iNOS) and cyclooxygenase-2 (COX-2) expression, cytokine interleukin-6 (IL-6) and tumor necrosis factor-α (TNF-α) production, and messenger RNA (mRNA) expression to attenuate inflammatory responses in lipopolysaccharide (LPS)-induced RAW 264.7 cells or stimulated bone marrow-derived macrophages (BMDMs). GH inhibited nuclear factor-κB (NF-κB) and mitogen-activated protein kinase (MAPK) pathways, the nuclear translocation of transcription factors NF-κB and activator protein 1 (AP-1), as well as upstream signaling of the toll-like receptor 4 (TLR4)-myeloid differentiation primary response 88 (MyD88) pathway in stimulated macrophages. Furthermore, the result of the intracellular signaling array showed that the phosphorylation of adenosine 5'-monophosphate-activated protein kinase-α (AMPKα), proline-rich Akt substrate of 40 kDa (PRAS40), and p38 could be down regulated by GH in BMDMs, indicating that the mechanism by which GH inhibited inflammation may be also associated with the energy metabolism pathway, PRAS40-mediated NF-κB pathway, cell proliferation, apoptosis, and autophagy, etc. In addition, GH alleviated dextran sodium sulfate (DSS)-induced colitis in mice by ameliorating weight loss, stool consistency change, blood in the stool, and colon shortening. GH decreased the protein and mRNA levels of IL-6 and TNF-α, iNOS and COX-2 mRNA expression, the activation of NF-κB and MAPK pathways, the phosphorylation of AMPKα and PRAS40, histological damage, and infiltration of macrophages in the colons of mice with DSS-induced colitis. Taken together, our results support that GH exerts the anti-inflammatory effects in macrophages *in vitro* through regulation of NF-κB and MAPK pathways, and DSS-induced colitis mouse model *in vivo.* These findings suggest that GH may be a promising candidate in treating macrophage-related inflammatory disease.

## Introduction

As one of the innate immune cells, macrophages play important roles in inflammation (Fujiwara and Kobayashi, 2005). Macrophages can be excessively activated by pathogen-associated molecular patterns (PAMPs) such as Gram-negative bacterial endotoxin lipopolysaccharide (LPS), and produce a cascade of inflammatory cytokines and mediators in response to LPS exposure (Hamidzadeh et al., 2017). Macrophages release various pro-inflammatory mediators including pro-inflammatory cytokines, oxygen and nitrogen species, such as nitric oxide (NO), tumor necrosis factor alpha (TNF-α), and interleukin-6 (IL-6), which finally lead to inflammatory response and tissue injury (Arango Duque and Descoteaux, 2014; Hamidzadeh et al., 2017). LPS can be recognized by toll-like receptor 4 (TLR4) on the cellular surface of macrophages (Aderem and Ulevitch, 2000), and then this interaction leads to the activation of several cellular signaling pathways involving nuclear factor-κB (NF-κB) and mitogen-activated protein kinases (MAPKs) signaling pathways, which is associated with the produce of inflammatory mediators (Guha and Mackman, 2001; Dong et al., 2002; Hamidzadeh et al., 2017). Macrophages are considered to be a potential therapeutic target in controlling many inflammatory diseases, such as inflammatory bowel disease (IBD) (Na et al., 2019).

Ulcerative colitis (UC), one form of IBD, is a recurrent chronic inflammatory disorder characterized by bloody diarrhea and the inflammation of colonic mucosa involving the activation of macrophages, dendritic cells, and T cells followed by the release of increased pro-inflammatory cytokines (Ordas et al., 2012). The current treatment of UC involves 5-aminosalicylic acid (5-ASA) drugs, corticosteroids, monoclonal antibodies against TNF-α, etc. However, a variety of side effects such as osteoporosis or myocarditis limits the utilization of these drugs (Talley et al., 2011).

Nowadays, many small molecular compounds from Chinese herbs have attracted more and more attention because of their inhibitory effects on inflammation (Wang et al., 2013). The genus *Garcinia* Linn. (Clusiaceae) is well known as a rich source of prenylated xanthones, polycyclic polyprenylated acylphloroglucinols (PPAPs), flavonoids, and polyisoprenylated benzophenones, which display a wide range of biological activities including antioxidative, antibacterial, anticancer, and anti-inflammatory effects (Kumar et al., 2013). *Garcinia oligantha* Merr. (Clusiaceae) is known to have traditional uses of cooling the inter-heat and detoxifying the body. This plant is also used to treat inflammatory diseases such as toothache, stomatitis, and scald (Libman et al., 2006; Gao et al., 2012). Gaudichaudione H (GH), a natural small molecular compound isolated from *G. oligantha* Merr. (Clusiaceae) has shown anti-cancer effects in several cancer cell lines such as HT-29, HeLa, and A549 cells (Gao et al., 2012; Tang et al., 2016). However, there is no study reporting its anti-inflammatory activities or the associated mechanisms to date. In this study, we investigated the anti-inflammatory effects and potential molecular mechanism of GH in LPS-induced macrophages *in vitro* and the dextran sodium sulfate (DSS)-induced colitis mouse model *in vivo.*

## Materials and Methods

### Materials

**Figure 1:**
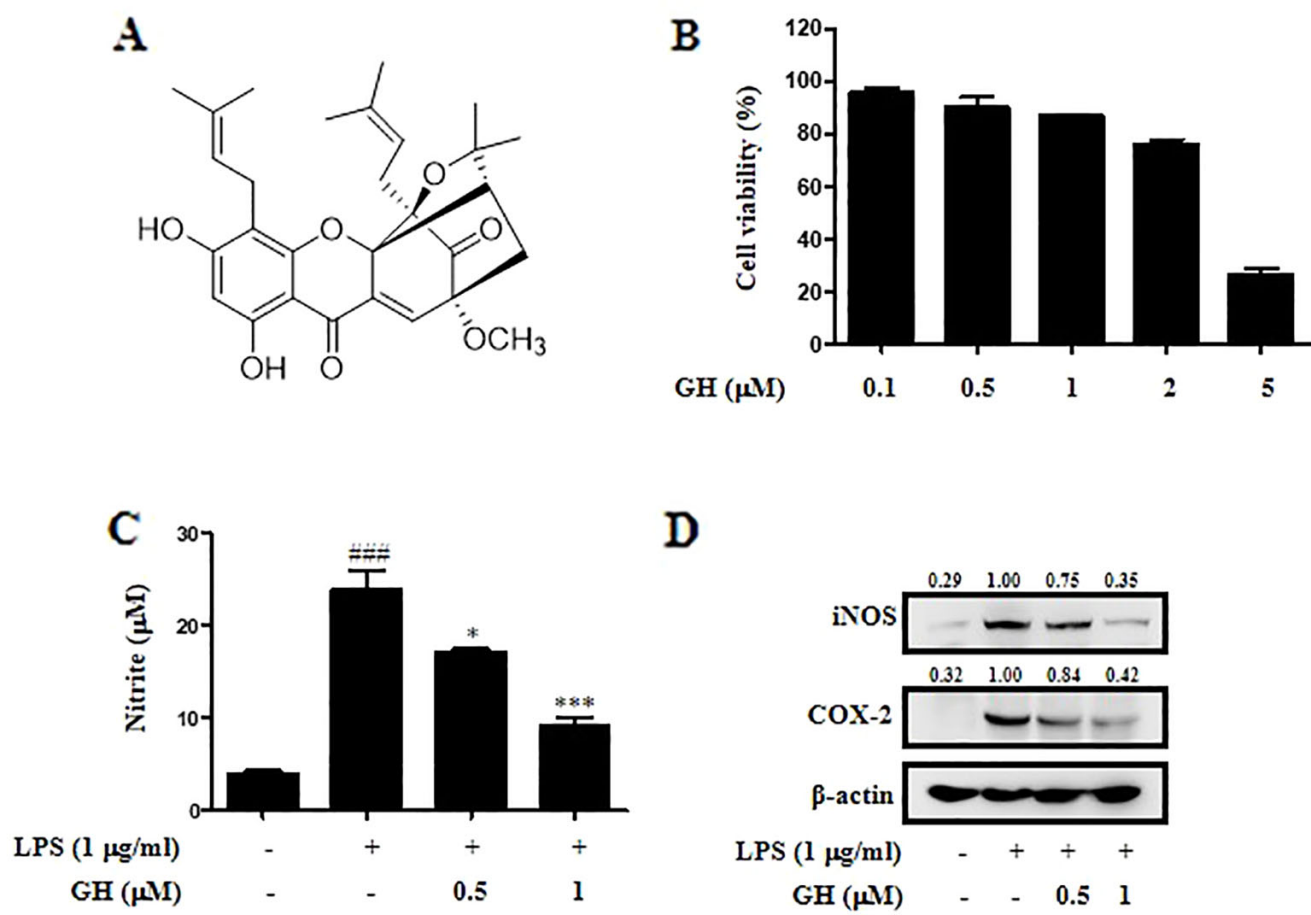
Gaudichaudione H (GH) inhibited nitric oxide (NO) production, inducible nitric oxide synthase (iNOS), and cyclooxygenase-2 (COX-2) expression in lipopolysaccharide (LPS)-induced RAW 264.7 cells. **(A)** Chemical structure of GH. **(B)** The effect of GH on cell viability. RAW 264.7 cells were treated with different concentrations of GH for 24 h. Cell viability was measured using the 2,5-diphenyltetrazolium bromide (MTT) assay. **(C)** The effect of GH on NO production. RAW 264.7 cells were treated with or without various concentrations of GH (0.5 and 1 μM) prior to LPS stimulation (1 μg ml^−1^). After 24 h, the supernatants were collected for NO measurement using Griess reagent. **(D)** GH inhibits iNOS and COX-2 expression in LPS-induced RAW 264.7 cells. Cells were incubated at 5×10^5^ cells well^−1^ in 12-well plates and treated with GH 1 h prior to LPS treatment. After 24 h, total protein lysate was collected for Western blot analysis. β-Actin was used as an internal control. The values above the bands mean the relative band intensities. Data were presented as the means ± SEM of three independent experiments. ^###^ *p* < 0.001 compared with control, *p < 0.05, ***p < 0.001 compared with LPS.

Gaudichaudione H (**Figure 1A**) was isolated from the *G. oligantha* Merr. (Clusiaceae). The leaves of *G. oligantha* Merr. (Clusiaceae) were collected in Hainan Province, China, in August 2013. The plant material was identified by Prof. Rongjing Zhang, South China Agricultural University. A voucher specimen (herbarium no. SHTYX-201309) was deposited at the Engineering Research Centre of Shanghai Colleges for TCM New Drug Discovery, Shanghai University of Traditional Chinese Medicine. The procedure of GH extraction was described previously (Tang et al., 2016). Gaudichaudione H's structure was determined using ^1^H-NMR and ^13^C-NMR spectral analysis, and the purity of this compound was more than 98% based on ultra performance liquid chromatography (UPLC) analysis (**Supplementary Figure 1**). GH was dissolved in dimethyl sulfoxide (DMSO). The final concentration of DMSO was adjusted to 0.1% (v/v) in culture media.

### Reagents

Dulbecco's modified Eagle's minimum essential medium (DMEM), fetal bovine serum (FBS), penicillin, and streptomycin were purchased from Gibco (Grand Island, NY, USA). *Escherichia coli* LPS, and 2,5-diphenyltetrazolium bromide (MTT) were obtained from Sigma Chemical Co. (St. Louis, MO, USA). Milli-Q water was supplied by a water purification system from Merck Millipore (Billerica, MA, USA). DSS (molecular weight of 36,000–50,000 Da) was obtained from MP Biomedicals (Irvine, CA, USA).

### Cell Culture and Treatment

The murine macrophage RAW 264.7 cell line was purchased from the Cell Bank of the Shanghai Institute of Cell Biology and Biochemistry, Chinese Academy of Sciences (Shanghai, China) and cultured in DMEM containing 10% heat-inactivated FBS, penicillin (100 U ml^−1^), and streptomycin (100 μg ml^−1^) at 37°C with 5% CO_2_. RAW 264.7 cells were incubated in the presence or absence of various concentrations of GH added 1 h prior to LPS (1 μg ml^−1^) treatment. Mouse fibroblast L929 cells were cultured in T75 flasks at a density of 1×10^5^ cells ml^−1^ and grown for 5 days to collect the supernatants. Bone marrow-derived macrophages (BMDMs) were isolated from male BALB/c mice aged 6–10 weeks and cultured in 12-well culture plates (5×10^5^ cells well^−1^) for use. Cells were differentiated in DMEM supplemented with 10% heat-inactivated FBS, penicillin (100 U ml^−1^), streptomycin (100 μg ml^−1^), and 70% supernatants from L929 cells for 7 days. The medium was changed on the following day and then changed once every 48 h and replaced with medium without supernatants from L929 cells at day 7. BMDMs were incubated in the presence or absence of various concentrations of GH added 4 h prior to LPS (10 μg ml^−1^) and interferon-γ (IFN-γ) (20 ng ml^−1^) treatment. DMSO alone was used as a vehicle in all *in vitro* experiments.

### Animals

BALB/c mice (male, 6–8 weeks of age, 18–20 g) were supplied from the Slac Animal Laboratory (Shanghai, China). The mice were kept under controlled room temperature, humidity, and light (12/12 h light/dark cycle) at Shanghai University of Traditional Chinese Medicine. All animal treatments complied with the animal ethics committee of Shanghai University of Traditional Chinese Medicine and were handled in accordance with the guidelines for Laboratory Animals Care and Usage of Shanghai University of Traditional Chinese Medicine (approval number SZY2018050071).

### Cell Viability

Cell viability was determined using the MTT assay. Briefly, RAW 264.7 cells were pre-incubated in 96-well plates at a density of 1 × 10^5^ cells ml^−1^ for 20 h and subsequently treated with various concentrations of GH (0.1, 0.5, 1, 2 and 5 μM). After culturing for 20 h, 20 μl of the MTT solution was added to each well and incubated for 4 h at 37°C and 5% CO_2_. A total of 150 μl of the supernatant was removed, and the formation of formazan was resolved with 150 μl of HCl-isopropanol solution. The absorption values were measured at 570 nm using a microplate reader. The cell viability was determined relative to the DMSO-treated cells.

### Measurement of Nitric Oxide Production

NO content was reflected by the amount of nitrite in the cell culture medium using the Griess Reagent System (Promega, Madison, WI, USA) according to the manufacturer's instructions. Briefly, RAW 264.7 cells and BMDMs were seeded in 12-well plates (5 × 10^5^ cells well^−1^) and treated with or without various concentrations of GH (0.5 and 1 μM) prior to stimulation. After 24 h, supernatants were collected, and a total of 50 μl of culture supernatants were incubated with 50 μl of 1% sulfanilamide in 5% phosphoric acid for 8 min at room temperature protected from light. Following that, 50 μl of 0.1% N-1-napthylethylenediamine dihydrochloride in water was added to all wells and the mixture was incubated for 8 min at room temperature. Absorbance was measured at 535 nm using a microplate reader, and the NO concentration was calculated based on a standard curve.

### Measurement of Interleukin-6 and Tumor Necrosis Factor-α Production

RAW 264.7 cells were cultured at 5×10^5^ cells well^−1^ in 12-well plates, and GH was pretreated 1 h prior to the stimulation by LPS (1 μg ml^−1^) for 24 h. After centrifugation at 10,000 rpm for 3 min, cell supernatants were collected. Colonic tissues were homogenized in radioimmunoprecipitation assay (RIPA) lysis buffer at 4°C, after centrifugation at 14,000 rpm for 15 min, supernatants were collected, and protein concentrations were quantified using bicinchoninic acid (BCA) reagents. Supernatants from RAW 264.7 cells and colon tissues were employed to detect levels of the pro-inflammatory cytokines IL-6 and TNF-α using enzyme-linked immunosorbent assay (ELISA) kits (DY406 and DY410, R&D Systems, Minneapolis, MN, USA), according to the manufacturer's instructions.

### Quantitative Real-Time Polymerase Chain Reaction

Total RNA was isolated from the cells and colon tissue homogenates using TRIzol (TaKaRa, Tokyo, Japan) according to the manufacturer's instructions and quantified using the spectrophotometer (DeNovix, Wilmington, Delaware, USA). The RNA was reverse transcribed into complementary DNA (cDNA) using a reverse transcription kit (Code No. RR047A, TaKaRa, Tokyo, Japan), and the gene amplification was proceeded by quantitative PCR analysis (SYBR Green; Applied Biosystems, Foster City, CA, USA). The PCR cycles were as follows: 95°C for 10 min, 40 cycles of 95°C for 15 s and 60°C for 60 s, and 1 cycle of 95°C for 15 s, 60°C for 60 s and 95°C for 15 s. Data were normalized to β-actin expression, and the results were analyzed using the 2^−ΔΔCt^ method. The primers used are listed as follows: mouse IL-6 forward, 5'-CTGCAAGAGACTTCCATCCAGTT-3', IL-6 reverse, 5'-GAAGTAGGGAAGGCCGTGG-3'; mouse TNF-α forward, 5'-CGAGTGACAAGCCTGTAGC-3', TNF-α reverse, 5'-GGTGTGGGTGAGGAGCACAT-3'; mouse inducible nitric oxide synthase (iNOS) forward, 5'-TCTTGGAGCGAGTTGTGGAT-3', iNOS reverse, 5'-TGACACAAGGCCTCCAATCT-3'; mouse cyclooxygenase-2 (COX-2) forward, 5'-GAAGTCTTTGGTCTGGTGCCT-3', COX-2 reverse, 5'-GCTCCTGCTTGAGTATGTCG-3'; mouse β-actin forward, 5'-GTATG-GAATCCTGTGGCATC-3', β-actin reverse, 5'-CGTACTCCTGCTTGCTGATC-3'.

### Western Blot

Cells were seeded at 5 × 10^5^ cells well^−1^ in 12-well plates and then stimulated with or without LPS (1 μg ml^−1^) or LPS (10 μg ml^−1^) together with IFN-γ (20 ng ml^−1^) for the indicated times in the presence or absence of different concentrations of GH. Cells were harvested and then lysed in RIPA lysis buffer [50 mM Tris pH 8.0, 150 mM NaCl, 1 mM ethylenediaminetetraacetic acid (EDTA), 1% Nonidet P-40, 0.5% sodium deoxycholate, 1 mM phenylmethanesulfonyl fluoride, 1% protease inhibitor cocktail, 1 mM NaF, and 1 mM Na_3_VO_4_]. After incubating on ice for 15 min, cell extracts were subjected to centrifugation at 14,000 rpm for 15 min at 4°C to get whole cell lysate protein. The cytoplasmic and nuclear extracts were prepared using Nuclear and Cytoplasmic Protein Extraction Kit (P0027, Beyotime Institute of Biotechnology, Jiangsu, China) according to the manufacturer's protocol. The protein concentration was measured using the BCA reagents. The amount of protein loaded on the gel is 20 μg. The protein samples underwent electrophoresis *via* sodium dodecyl sulfate-polyacrylamide gel electrophoresis (SDS-PAGE) and then transferred onto nitrocellulose membrane. The membranes were blocked with 5% nonfat dry milk in Tris-buffered saline with 0.1% Tween 20 (TBST) for 2 h and incubated with primary antibodies at 4°C overnight. Primary antibodies used (1:500–10,000 dilution) were as follows: antibodies against iNOS (#13120), COX-2 (#4842), phospho-IκB kinase α/β (p-IKKα/β) (#2697), IKKα/β (#3700), phospho-IκBα (#2859), IκBα (#4812), phospho-extracellular signal-regulated kinase (p-ERK) ½ (#4370), ERK ½ (#9102), phospho-p38 (#4511), p38 (#8690), p65 (#8242), p-c-Jun (#3270), c-Jun (#9165), c-fos (#2250), β-tubulin (#2146), Lamin A/C (#4777), phospho-c-Jun N-terminal kinase (p-JNK) (#4668), JNK (#9252), myeloid differentiation primary response 88 (MyD88) (#4283), phospho-IL-1 receptor-associated kinase 4 (p-IRAK4) (#11927), IRAK4 (#4363), phospho-adenosine 5'-monophosphate-activated protein kinase-α (p-AMPKα) (#50081), AMPKα (#5831), phospho-proline-rich Akt substrate of 40 kDa (p-PRAS40) (#13175), PRAS40 (#2610), and β-actin (#4970) (Cell Signaling Technology, Danvers, MA, USA) and the antibody against TLR4 (sc-293072, Santa Cruz Biotechnology, Santa Cruz, CA, USA). Membranes were washed three times with TBST buffer. After incubation for 1.5 h with horseradish peroxidase-conjugated goat anti-rabbit or anti-mouse immunoglobulin G (IgG) at dilutions of 1:2,500, membranes were again washed three times with TBST buffer, and then developed using electrochemiluminescence (ECL) detection kits (36208ES60, Yeasen Biotech, Shanghai). Integrated densities of bands were quantified using ImageJ software. The ponceau staining and the β-actin as loading control were showed in **Supplementary Figure 3**.

### Immunofluorescence

RAW 264.7 cells were seeded at 3 × 10^5^ cells well^−1^ on sterile coverslips in 12-well plates, precultured with GH for 1 h, and then incubated in the presence or absence of LPS for 30 min. Cells were washed with phosphate buffered saline (PBS) and fixed with 4% paraformaldehyde for 15 min and permeabilized with 0.1% Triton X-100 for 10 min at room temperature. Cells were washed, blocked with 3% bovine serum albumin (BSA) in PBS for 1 h, and then incubated with p65 (1:400 in 3% BSA)-, p-c-Jun (1:100 in 3% BSA)-, or c-fos (1:6,400 in 3% BSA)-specific antibodies overnight at 4°C. The slides were then washed three times and incubated with fluorescein isothiocyanate (FITC)-conjugated anti-rabbit IgG antibody for 1.5 h at room temperature. After three times of washing, 4',6-diamidino-2-phenylindole (DAPI) was added for 2 h in the dark for nuclear staining. Images were captured using a fluorescence microscope at 60× magnification.

### Pathscan^®^ Intracellular Signaling Array

BMDMs were cultured in 12-well plates and pretreated with GH for 4 h and then stimulated with LPS (10 μg ml^−1^) and IFN-γ (20 ng ml^−1^). Cell lysates were prepared to detect intracellular signaling molecules using a PathScan^®^ intracellular signaling array kit (Cell Signaling Technology, Danvers, MA, USA, #7323) according to the manufacturer's procedure.

### Dextran Sodium Sulfate-Induced Colitis

The mice were acclimated for 1 week before the experiments. The mice were weighed and randomly separated into four experimental groups (n = 10/group) as follows: group 1 was the control group allowed plain drinking water *ad libitum*; the animals in group 2 (3.5% DSS group), group 3 (GH 10 mg kg^−1^ group), and group 4 (GH 20 mg kg^−1^ group) were treated with 3.5% DSS in the drinking water with or without intragastric administration of GH (0.2 ml 10 g^−1^) once a day for 7 consecutive days. GH was dissolved in 0.9% physiological saline containing 0.5% Tween 80 which was administered to the control group and the 3.5% DSS group. During the experimental period, the development of clinical symptoms (weight loss, severity of diarrhea, and rectal bleeding) of colitis was recorded daily. The disease activity index (DAI) value was determined by combining scores of weight loss (0, none; 1, 0–5%; 2, 5–10%; 3, 10–20%; 4, > 20%), stool consistency change (0, none; 1 and 2, loose stool; 3 and 4, diarrhea), and bleeding (0, none; 1, trace; 2, mild bleeding; 3, obvious bleeding; 4, gross bleeding), and then dividing by 3 (Wen et al., 2013). The minimal score was 0, and the maximal score was 4. The mice were sacrificed on day 7, and colon tissues were collected for ELISA, hematoxylin and eosin (H&E) staining, RT-PCR, Western blot and immunohistochemistry (IHC). The colon length was measured between the ileo-cecal junction and the proximal rectum. Colon tissues were free of adherent adipose tissue, rinsed with saline to remove fecal residue.

### Microscopic Scoring of Colonic Injury

The colon was fixed in 4% phosphate-buffered paraformaldehyde, embedded in paraffin, cut into sections, and then placed on microscope slides. Slides were stained with H&E and observed using DP-72 microscope (Olympus, Tokyo, Japan) to evaluate histological damage. Assessment included reporting of edema, extent of injury, and crypt abscesses. The grading system was divided into three categories as follows: inflammation severity (0, no inflammation; 1, slight inflammation; 2, moderate inflammation; and 3, severe inflammation), the extent of injury (0, no injury; 1, mucosal injury; 2, mucosal and submucosal injury; and 3, transmural injury), and crypt damage (0, no damage; 1, basal third was damaged; 2, basal two-thirds was damaged; and 3, only the surface epithelium was intact; 4, loss of entire crypt and epithelium). The final histological damage score was the sum of each parameter (Lee et al., 2015).

### Immunohistochemistry Staining

For immunohistochemical staining, mouse anti-rabbit F4/80 (GB11027, Servicebio, Hubei, China, 1:500 dilution) and mouse anti-rabbit CD68 (GB11067, Servicebio, Hubei, China, 1:500 dilution) were used as the primary antibodies and goat anti-rabbit IgG as the secondary antibody (GB23303, Servicebio, Hubei, China, 1:200 dilution). IHC was performed using standard protocols (Leonhardt et al., 2003). Negative control sections were treated in the same way, omitting primary antibodies. Images were captured using DP-72 microscope (Olympus, Tokyo, Japan). The expression levels were quantified by Image Pro-Plus v.6.0 software *via* analyzing the mean value of integral optical density (IOD).

### Statistical Analysis

Results were presented as the means ± SEM. All data were from three independent experiments. Statistical analysis was performed using GraphPad Prism software 5.0 (San Diego, CA, USA). The differences between two groups were analyzed using the unpaired Student's t test and those between multiple groups were assessed using one-way analysis of variance (ANOVA) with Tukey's multiple comparison test, and *p*-values of less than 0.05 were considered statistically significant.

## Results

### Gaudichaudione H Inhibited Nitric Oxide Production in Lipopolysaccharide-Induced RAW 264.7 Cells

First, the effect of GH on cell viability was evaluated using the MTT assay after 24 h of GH treatment (**Figure 1B**). The results showed that GH did not affect cell viability of RAW 264.7 macrophages when cells were treated with 0.1, 0.5, or 1 μM GH. We also performed propidium iodide (PI)/annexin V analysis to evaluate the effect of GH on the cell state at different time points (1, 4, 12, 24 h). The results showed that GH did not affect the cell viability of Raw 264.7 cells at different time points (**Supplementary Figure 2**). Here, we detected NO production in activated RAW 264.7 cells to assess the potential anti-inflammatory effect of GH. GH decreased LPS-induced NO production in RAW 264.7 cells in a concentration-dependent manner (**Figure 1C**). NO is mainly generated from L-arginine in macrophages by iNOS, and another enzyme COX-2 is also an important inflammatory mediator which induces the production of prostaglandin E2 (PGE2) (Kam and See, 2000; Aktan, 2004). In addition, it is reported that iNOS is inducible by COX-2 (Chiarugi et al., 1998). We measured the expression of iNOS and COX-2, and found that the levels of iNOS and COX-2 in LPS-induced RAW 264.7 cells were reduced by GH in a dose-dependent manner (**Figure 1D**). The data showed that GH could suppress NO production, iNOS and COX-2 expression in LPS-induced RAW 264.7 cells without affecting cell viability.

### Gaudichaudione H Inhibited Interleukin-6 and Tumor Necrosis Factor-α Production and Messenger Ribonucleic Acid Expression in Lipopolysaccharide-Stimulated RAW 264.7 Cells

**Figure 2:**
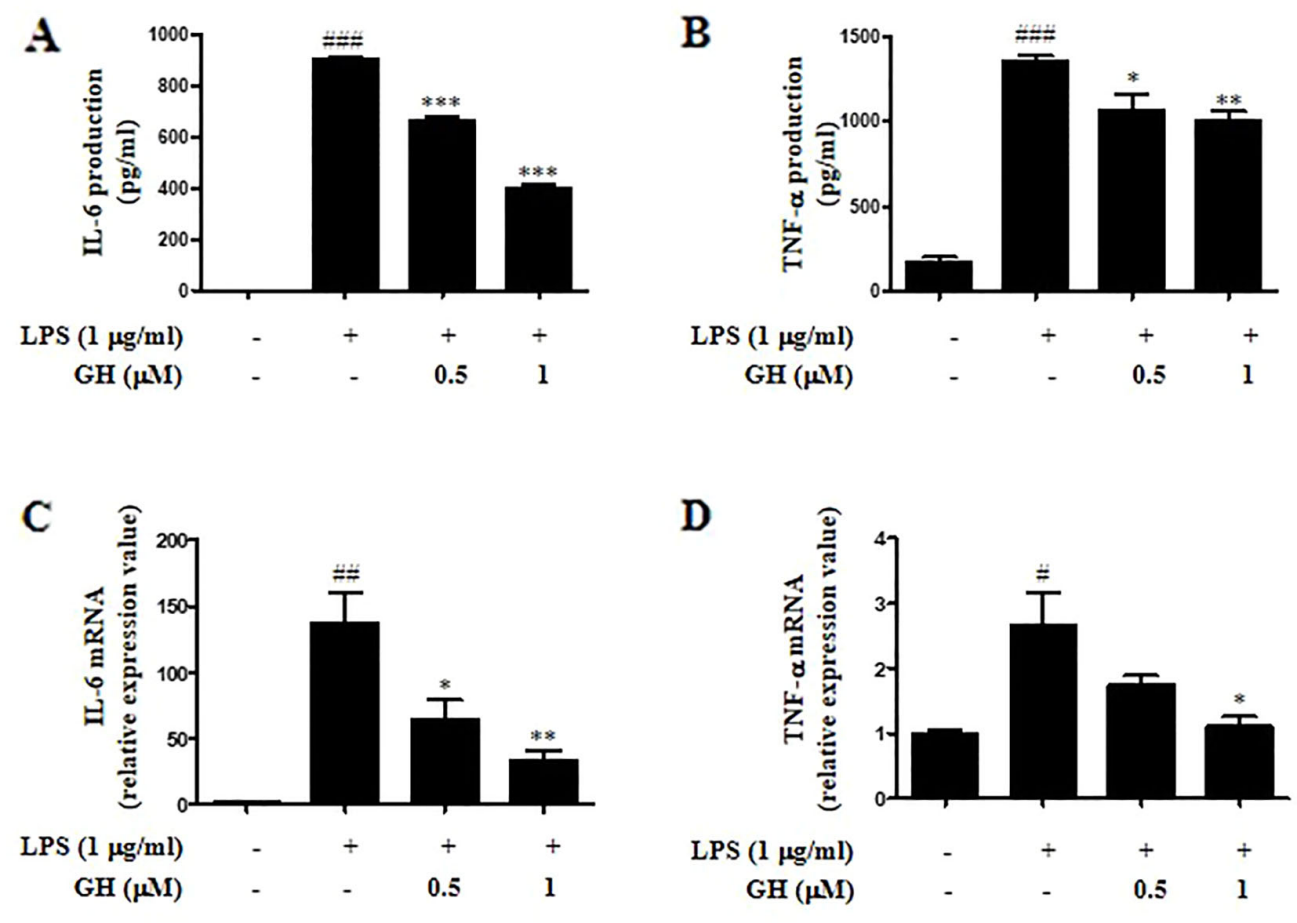
Gaudichaudione H (GH) inhibited cytokine interleukin-6 (IL-6) and tumor necrosis factor-α (TNF-α) production and messenger RNA (mRNA) expression in lipopolysaccharide (LPS)-stimulated RAW 264.7 cells. RAW 264.7 cells were cultured at 5×10^5^ cells well^−1^ in 12-well plates, and GH was pretreated for 1 h and then co-treated with LPS (1 μg ml^−1^) for 24 or 6 h. After 24 h of stimulation, IL-6 **(A)** and TNF-α **(B)** in the cell supernatants were assayed using ELISA. After 6 h of stimulation, the cells were collected, and the mRNA expressions of IL-6 **(C)** and TNF-α **(D)** were detected using real-time PCR (RT-PCR). Data were presented as the means ± SEM of three independent experiments. ^#^ *p* < 0.05, ^##^ *p* < 0.01, ^###^ *p* < 0.001 compared with control, *p < 0.05, **p < 0.01, ***p < 0.001 compared with LPS.

Since both IL-6 and TNF-α are important pro-inflammatory cytokines in the modulation of inflammation (Arango Duque and Descoteaux, 2014), we detected both protein and messenger RNA (mRNA) levels of IL-6 and TNF-α using ELISA and quantitative real-time polymerase chain reaction (qRT-PCR). The results displayed that GH dose-dependently inhibited the secretion of inflammatory cytokines IL-6 and TNF-α after 24 h of LPS stimulation in RAW 264.7 cells (**Figures 2A, B**) and showed a similar inhibitory effect on the mRNA expression of IL-6 and TNF-α after 6 h of LPS stimulation (**Figures 2C, D**). In addition, GH inhibited cytokine IL-6 and TNF-α production when RAW 264.7 cells were first treated with LPS for 1 h before adding GH, indicating that GH could rescue LPS-induced inflammation in RAW 264.7 cells (**Supplementary Figure 4**).

### Gaudichaudione H Inhibited Nuclear Factor-κB and Mitogen-Activated Protein Kinase Pathways Activation in RAW 264.7 Cells

**Figure 3:**
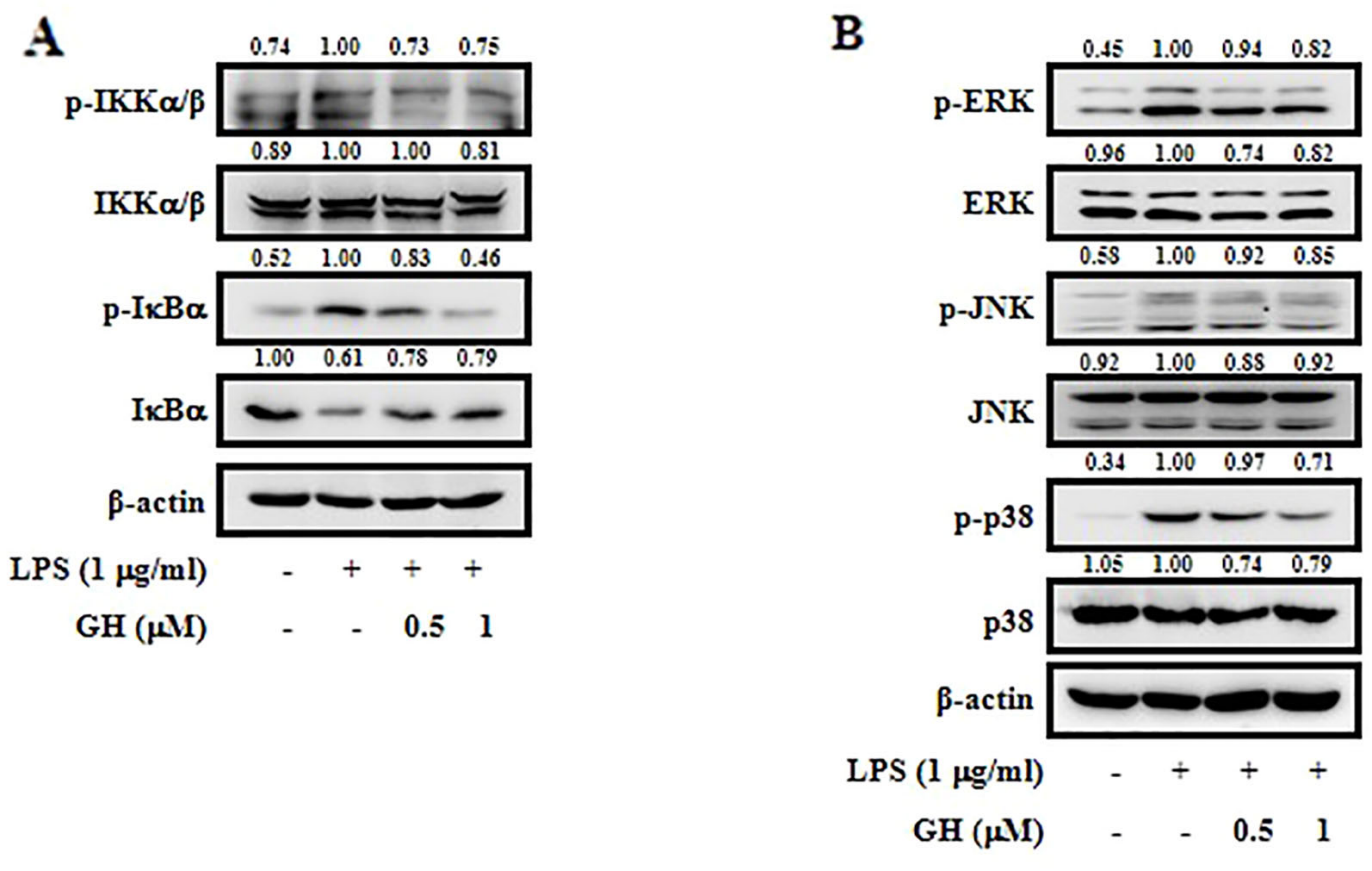
Gaudichaudione H (GH) inhibited nuclear factor-κB (NF-κB) and mitogen-activated protein kinase (MAPK) pathways in lipopolysaccharide (LPS)-stimulated RAW 264.7 cells. Cells were pretreated with GH for 1 h prior to LPS treatment (1 μg ml^−1^). The levels of phospho-IκB kinase α/β (p-IKKα/β), IKKα/β, phospho-IκBα (p-IκBα), and IκBα were measured after 15 min of LPS treatment. The levels of phospho-extracellular signal-regulated kinase (p-ERK), ERK, phospho-c-Jun N-terminal kinase (p-JNK), JNK, phospho-p38 (p-p38), and p38 were measured after 30 min of LPS treatment. The signaling molecules in the NF-κB pathway **(A)** and the MAPK pathway **(B)** were analyzed using Western blot. The values above the bands mean the relative band intensities. β-Actin was used as an internal control. Data were from three independent experiments.

It is well-known that NF-κB is an important signaling molecule in the development of inflammatory diseases. In its inactive form, NF-κB exists in the cytoplasm and binds to its inhibitor IκB (Park et al., 2017). Upon activation by LPS, IκB is phosphorylated by IKKα/β, leading to its degradation and NF-κB activation (Qi et al., 2012). To investigate whether the inhibition of the inflammatory response by GH is mediated through the NF-κB pathway, we examined p-IKKα/β, IKKα/β, p-IκBα, and IκBα expression in LPS-induced RAW 264.7 cells. The results showed that GH inhibited LPS-induced phosphorylation of IKKα/β and IκBα as well as degradation of IκBα (**Figure 3A**). MAPKs, including ERK, JNK, and p38, also play critical roles in regulating inflammatory gene expression and cytokine production (Lai et al., 2017). We measured the effect of GH on MAPKs and found that GH down regulated the expression of phosphorylated ERK, JNK, and p38 in LPS-induced RAW 264.7 cells in a dose-dependent manner (**Figure 3B**). Taken together, these findings indicated that GH showed a potential anti-inflammatory effect *via* the suppression of NF-κB and MAPK pathways activation.

### Gaudichaudione H Inhibited Nitric Oxide Production, Nuclear Factor-κB, and Mitogen-Activated Protein Kinase Pathways in Activated Bone Marrow-Derived Macrophages

**Figure 4:**
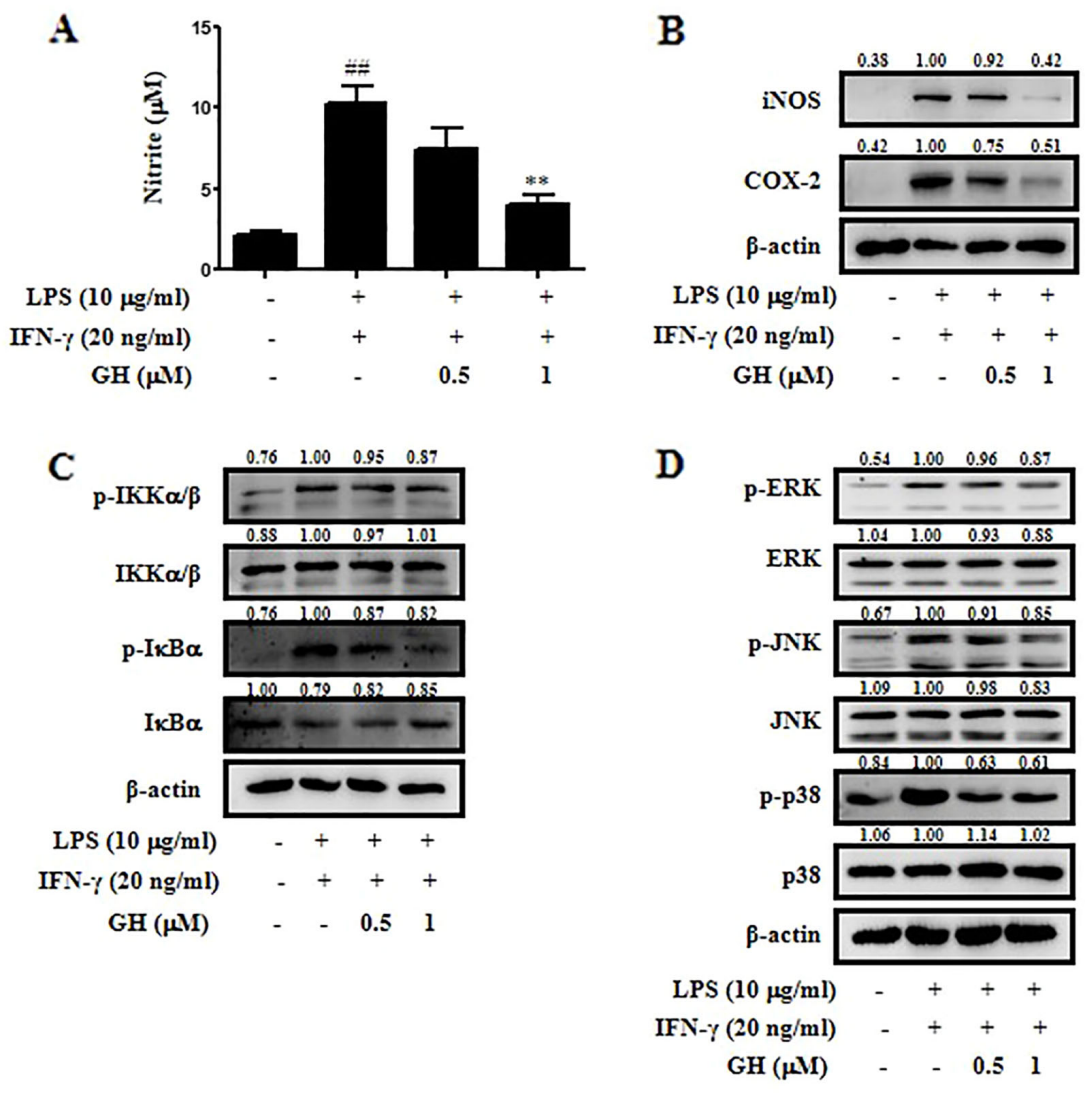
Gaudichaudione H (GH) inhibited nitric oxide (NO) production, nuclear factor-κB (NF-κB) pathway, and mitogen-activated protein kinase (MAPK) pathway in stimulated bone marrow-derived macrophages (BMDMs). BMDMs were cultured in 12-well plates and pretreated with GH for 4 h and then stimulated with lipopolysaccharide (LPS) (10 μg ml^−1^) and interferon-γ (IFN-γ) (20 ng ml^−1^). **(A)** NO production was measured using Griess reagent. **(B)** The expression of inducible nitric oxide synthase (iNOS) and cyclooxygenase-2 (COX-2) were detected using Western blot. The protein levels of the signaling molecules in the NF-κB pathway **(C)** and the MAPK pathway **(D)** were analyzed using Western blot. β-Actin was used as an internal control. The values above the bands mean the relative band intensities. Data were presented as the means ± SEM of three independent experiments. ^##^ *p* < 0.01 compared with control, **p < 0.01 compared with LPS and IFN-γ.

We used primary murine BMDMs to further determine the anti-inflammatory effects of GH on macrophages. The results displayed the inhibitory effect of GH in BMDMs similar to that in RAW 264.7 cells. GH inhibited NO production (**Figure 4A**), iNOS and COX-2 expression (**Figure 4B**), as well as NF-κB and MAPK pathways (**Figures 4C, D**) in BMDMs stimulated by LPS (10 μg ml^−1^) and IFN-γ (20 ng ml^−1^).

### Gaudichaudione H Inhibited Nuclear Translocation of Nuclear Factor-κB and Activator Protein 1 in Lipopolysaccharide-Induced RAW 264.7 Cells

**Figure 5:**
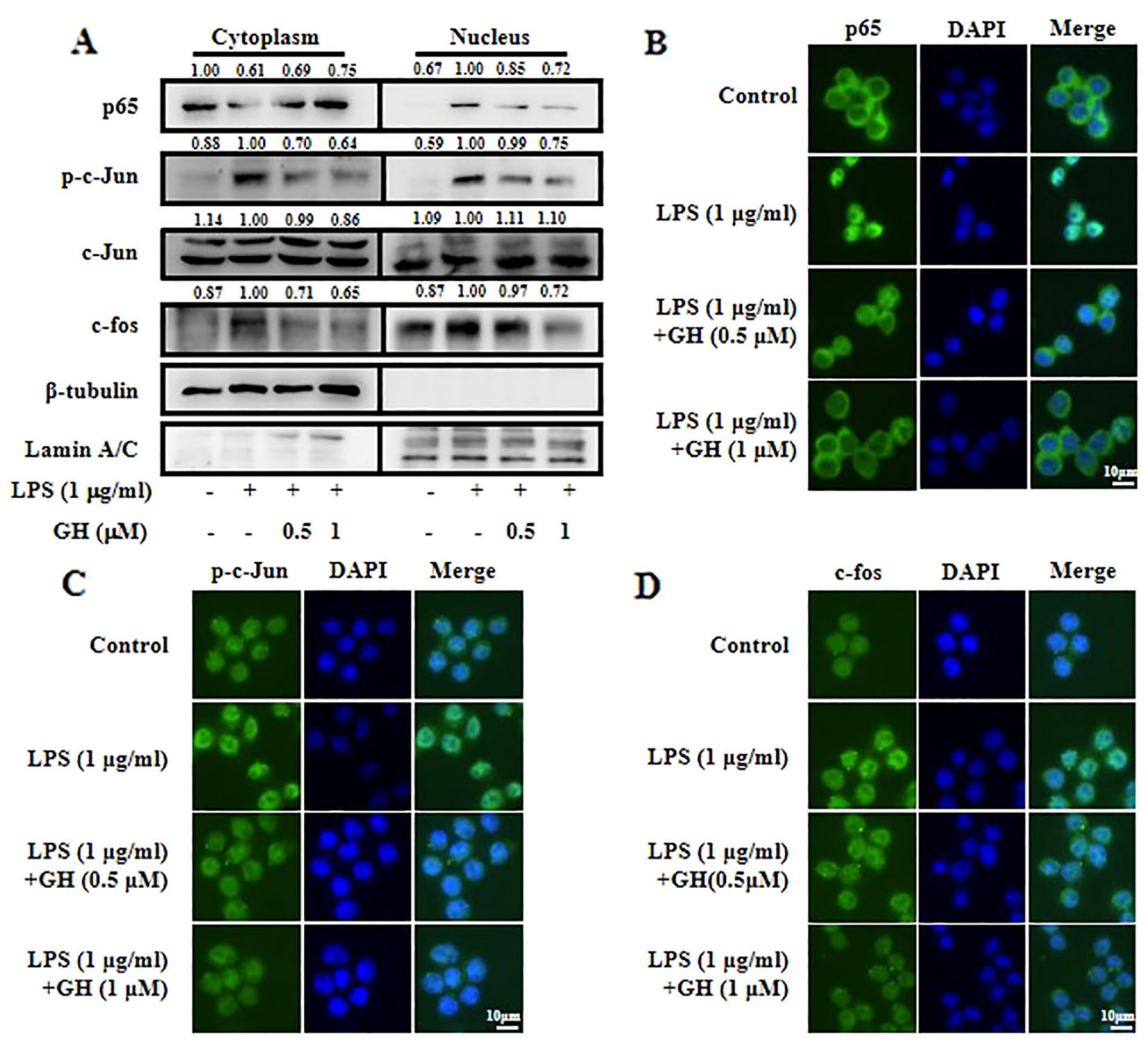
Gaudichaudione H (GH) inhibited the nuclear translocation of nuclear factor-κB (NF-κB) and activator protein 1 (AP-1) in lipopolysaccharide (LPS)-stimulated RAW 264.7 cells. **(A)** Cells were collected after pre-incubation with GH for 1 h and subsequent treatment with 1 μg ml^−1^ LPS for 30 min. The cytoplasmic and nuclear extracts lysates were prepared using the Nuclear and Cytoplasmic Protein Extraction Kit (P0027, Beyotime Biotechnology) according to the manufacturer's protocol. The proteins located in the cytoplasm or nucleus were determined by Western blot analysis. The values above the bands mean the relative band intensities. β-Tubulin and Lamin A/C were used as internal controls. Furthermore, cellular localizations of p65 **(B)**, p-c-Jun **(C)**, and c-fos **(D)** were determined by immunofluorescence. Data were from three independent experiments.

Two transcription factors, NF-κB and activator protein 1 (AP-1), could translocate from the cytoplasm into the nucleus and enhance the transcription of inflammatory genes such as iNOS, COX-2, IL-6, and TNF-α to mediate the initiation and amplification of inflammation in response to stimulation (Fujioka et al., 2004; Hoesel and Schmid, 2013). Transcription factor p65 is a major functional subunit of NF-κB, and AP-1 consists of the Jun and Fos families, which are modulated by the MAPK signaling pathway (Kaminska, 2005). Therefore, we used Western blot and immunofluorescence to assess the ability of GH to inhibit nuclear translocation of NF-κB and AP-1 in LPS-induced RAW 264.7 cells. We found that nuclear translocation of NF-κB and AP-1 was decreased following GH treatment in a dose-dependent manner (**Figure 5A**). The results of immunofluorescence also demonstrated that GH inhibited the nuclear translocation of p65 (**Figure 5B**), p-c-Jun (**Figure 5C**), and c-fos (**Figure 5D**).

### Gaudichaudione H Suppressed the Toll-Like Receptor 4-Myeloid Differentiation Primary Response 88 Pathway in Activated RAW 264.7 Cells and Bone Marrow-Derived Macrophages

**Figure 6:**
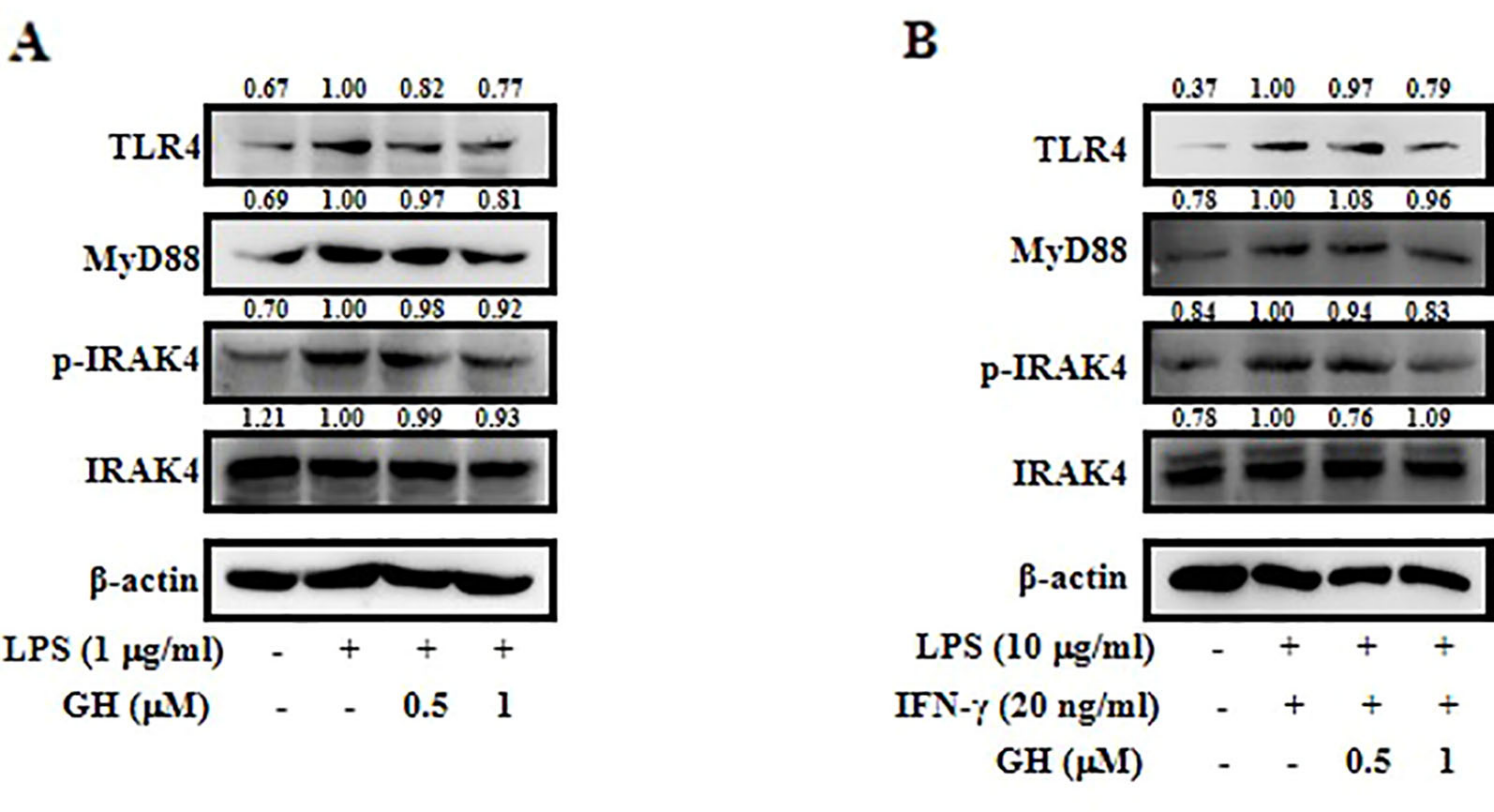
Gaudichaudione H (GH) suppressed the toll-like receptor 4 (TLR4)-myeloid differentiation primary response 88 (MyD88) pathway in activated RAW 264.7 cells and bone marrow-derived macrophages (BMDMs). Cells were pretreated with GH and then stimulated for 15 min in RAW 264.7 cells **(A)** and BMDMs **(B)**. Protein samples were analyzed using Western blot with specific antibodies. The values above the bands mean the relative band intensities. β-Actin was used as an internal control. Data were from three independent experiments.

TLR4 is the major upstream signaling receptor for LPS by activating downstream signaling pathways including the MAPK and NF-κB pathways (Kagan and Medzhitov, 2006). To test whether GH affects TLR4-mediated signaling, the protein levels of TLR4, MyD88, IRAK4, and p-IRAK4 were measured in both RAW 264.7 cells (**Figure 6A**) and BMDMs (**Figure 6B**) using Western blot. In addition, the qRT-PCR data showed a similar inhibitory effect on the mRNA expression of TLR4 and MyD88 in RAW 264.7 cells (**Supplementary Figure 5**). The results showed that GH downregulated the activation of TLR4-MyD88 signaling pathway in RAW 264.7 cells and BMDMs.

### Gaudichaudione H Blocked Adenosine 5'-Monophosphate-Activated Protein Kinase-α, Proline-Rich Akt Substrate of 40 kDa, and P38 Phosphorylation in Bone Marrow-Derived Macrophages

**Figure 7:**
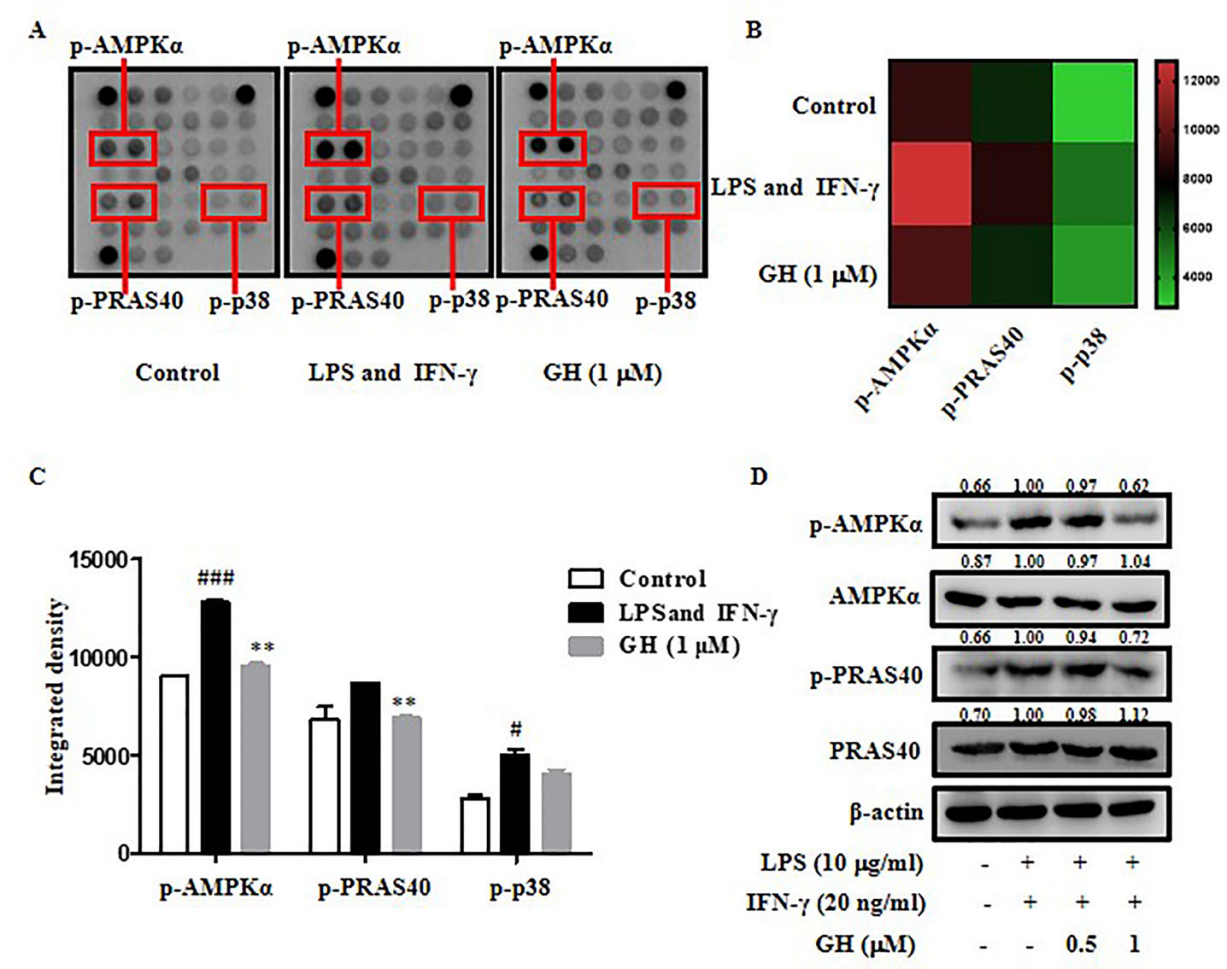
Gaudichaudione H (GH) blocked adenosine 5'-monophosphate-activated protein kinase-α (AMPKα), proline-rich Akt substrate of 40 kDa (PRAS40), and p38 phosphorylation in bone marrow-derived macrophages (BMDMs). **(A)** The phosphorylation of AMPKα, PRAS40, and p38 were down regulated by GH in BMDMs, as detected by the intracellular signaling antibody array. **(B, C)** A heat map and diagram of the changes were shown. **(D)** Western blot analysis of phosphorylated AMPKα and PRAS40 and respective total protein in BMDMs is shown. The values above the bands mean the relative band intensities. β-Actin was used as an internal control. Data were presented as the means ± SEM of three independent experiments. ^#^ *p* < 0.05, ^###^ *p* < 0.001 compared with control, **p < 0.01 compared with LPS and IFN-γ.

To further illuminate the molecular mechanisms by which GH regulates inflammation, a PathScan^®^ Intracellular Signaling Array was used to detect the changes in 18 important signaling molecules that are phosphorylated or cleaved in response to signal-transduction pathway activation which are associated with inflammation, energy metabolism, apoptosis, etc. We found that the phosphorylation of AMPKα, PRAS40, and p38 was down regulated by GH in BMDMs induced by LPS and IFN-γ (**Figures 7A–C**). The data indicated that GH exerted anti-inflammatory effects *via* the blockade of AMPKα, PRAS40, and p38 activations. Since p38 phosphorylation in macrophages has been detected by Western blot before, we further examined phosphorylation of other two proteins, and the results showed a similar inhibitory effect of GH on AMPKα and PRAS40 phosphorylation (**Figure 7D**). PRAS40 could modulate the NF-κB pathway and elevate NF-κB transcriptional activity (Zhu et al., 2017). AMPKα is an important molecule regulating energy metabolism and closely related to energy and substance metabolism, cell proliferation, apoptosis, autophagy, etc. (Ye, 2013). The results indicated that the mechanism by which GH inhibited inflammation may be also associated with the energy metabolism pathway, PRAS40 mediated NF-κB pathway, cell proliferation, apoptosis, autophagy, etc.

### Gaudichaudione H Alleviated Symptoms of Dextran Sodium Sulfate-Induced Colitis in Mice

**Figure 8:**
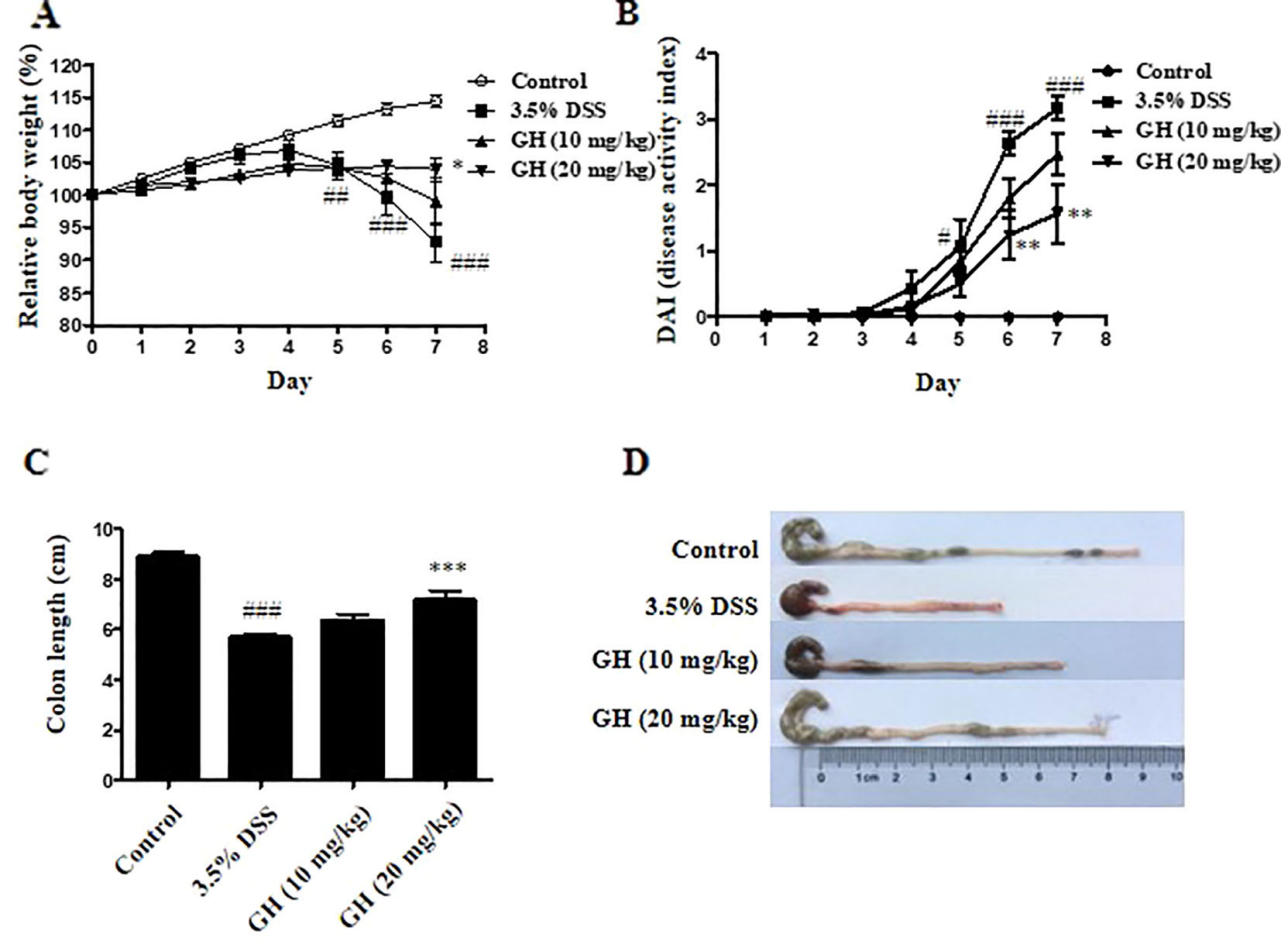
Gaudichaudione H (GH) alleviated symptoms of dextran sodium sulfate (DSS)-induced colitis in mice. BALB/c mice were administered with 3.5% DSS in drinking water and treated with or without the intragastric administration of GH (10 or 20 mg kg^−1^). **(A)** The relative body weight of each mouse was measured. **(B)** Disease activity index. **(C, D)** Analysis of the colon length from the experimental mouse groups (n = 10/group). The data are the means ± SEM, n = 10. ^#^ *p* < 0.05, ^##^ *p* < 0.01, ^###^ *p* < 0.001 compared with control, *p < 0.05, **p < 0.01, ***p < 0.001 compared with 3.5% DSS.

First, we performed toxicity experiment of GH in mice before the mouse model of DSS-induced colitis. As indicated in Supplementary Materials (**Supplementary Figure 6**), the results showed that there were no significant morphologic differences including the shape, size, and color in the liver, heart, spleen, lung, and kidney tissues of the mice of the GH group compared with the tissues of the control group mice. The results showed that GH at a concentration of 20 mg kg^−1^ or less did not cause tissues damage and had no toxic effect on mice. Therefore, we chose two concentrations of 10 and 20 mg kg^−1^ as the concentration of GH administered in the mouse model. The DSS-induced colitis model is well-established and widely used in the study of IBD pathogenesis and preclinical studies (Perse and Cerar, 2012). We use this model to further examine the anti-inflammatory effect of GH *in vivo*. We observed symptoms of weight changes, stool consistency change, hematochezia, and colon length changes to assess the severity of DSS-induced colitis. The data showed that the 3.5% DSS group had significant weight loss beginning on day 5, and GH alleviated weight loss at day 7 (**Figure 8A**). The DAI value increased gradually and reached its maximum on day 7. GH significantly reduced the DAI score in a dose-dependent manner, comparing with the increased DAI value in the 3.5% DSS group (**Figure 8B**). The colons of mice in the 3.5% DSS group were significantly shortened by DSS, and a high dose of GH obviously attenuated colitis-induced colon length reduction (**Figures 8C, D**).

### Gaudichaudione H Inhibited Cytokines Production and Inflammatory Genes Messenger Ribonucleic Acid Expression in Colon of Dextran Sodium Sulfate-Induced Mice

**Figure 9:**
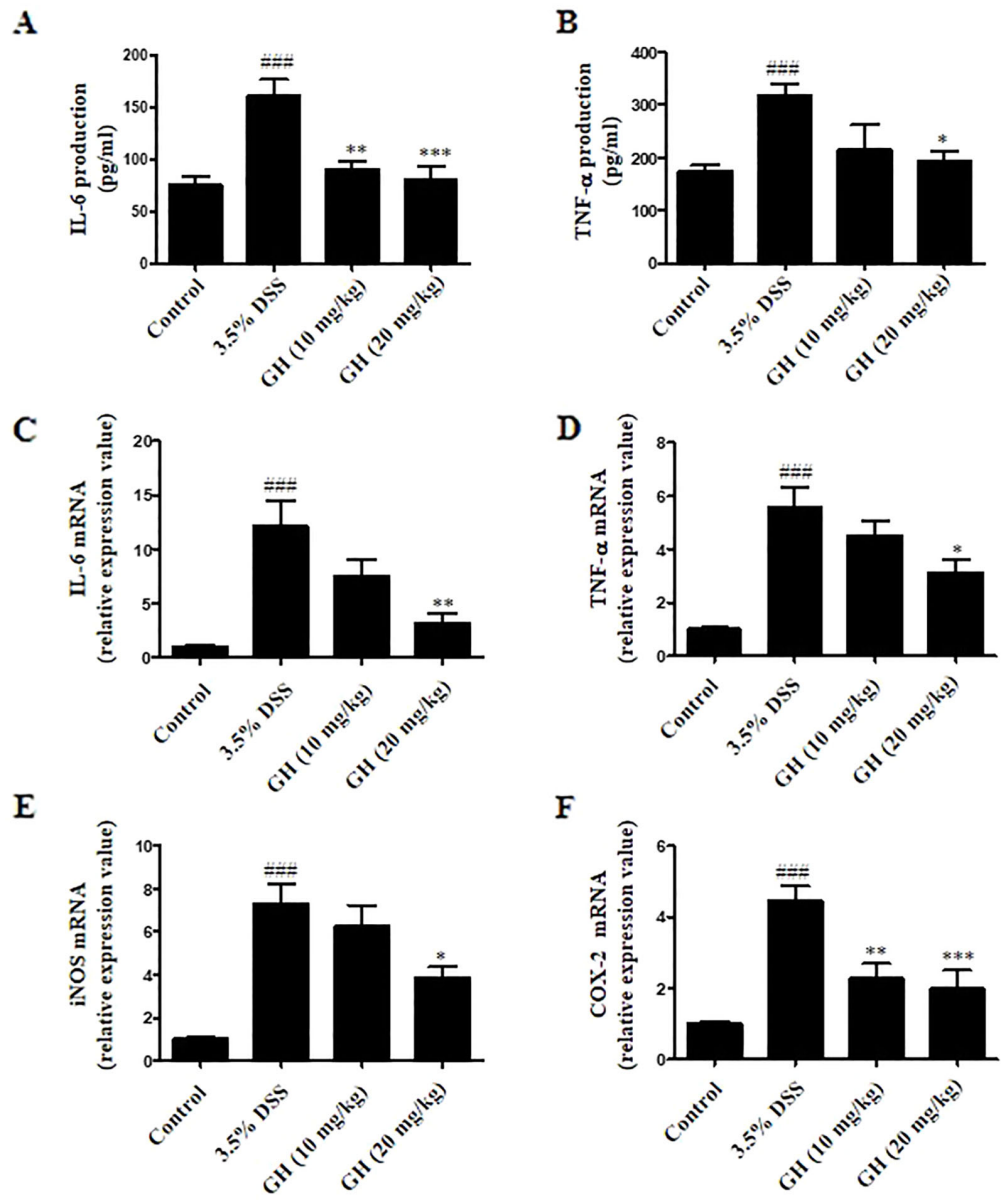
Gaudichaudione H (GH) inhibited cytokines production and inflammatory genes messenger RNA (mRNA) expression in colon of dextran sodium sulfate (DSS)-induced mice. Colons from mice were removed at the end of experiment, and finally lysed. Interleukin-6 (IL-6) **(A)** and tumor necrosis factor-α (TNF-α) **(B)** production in the supernatants were assayed using ELISA. The data are the means ± SEM, n = 7. Total RNA from colon tissues was isolated using TRIzol reagent, the mRNA expressions of IL-6 **(C)**, TNF-α **(D)**, inducible nitric oxide synthase (iNOS) **(E)**, and cyclooxygenase-2 (COX-2) **(F)** were detected using real-time PCR (RT-PCR). The data are the means ± SEM, n = 8. ^###^ *p* < 0.001 compared with control, *p < 0.05, **p < 0.01, ***p < 0.001 compared with 3.5% DSS.

We evaluated levels of inflammation in the colon tissue by detecting IL-6, TNF-α, iNOS, and COX-2 mRNA expression, as well as IL-6 and TNF-α production from colonic tissue. As shown in **Figures 9A, B**, the levels of pro-inflammatory cytokines IL-6 and TNF-α in colon tissues were elevated in DSS-treated mice in comparison with those of the control group, and GH could effectively decrease the production of IL-6 and TNF-α in the colons. In addition, significant decrease in mRNA levels of IL-6 (**Figure 9C**), TNF-α (**Figure 9D**), iNOS (**Figure 9E**), and COX-2 (**Figure 9F**) in the colon tissues was observed in the GH (20 mg kg^−1^) group, comparing with the 3.5% DSS group. On the basis of these observations, it was indicated that GH treatment could effectively attenuate the abnormity of inflammatory cytokines and inflammatory gene expression in the colons of mice with DSS-induced colitis.

### Gaudichaudione H Inhibited Nuclear Factor-κB/Mitogen-Activated Protein Kinase Pathway and the Phosphorylation of Adenosine 5'-Monophosphate-Activated Protein Kinase-α and Proline-Rich Akt Substrate of 40 kDa in Colonic Tissues in Mice

**Figure 10:**
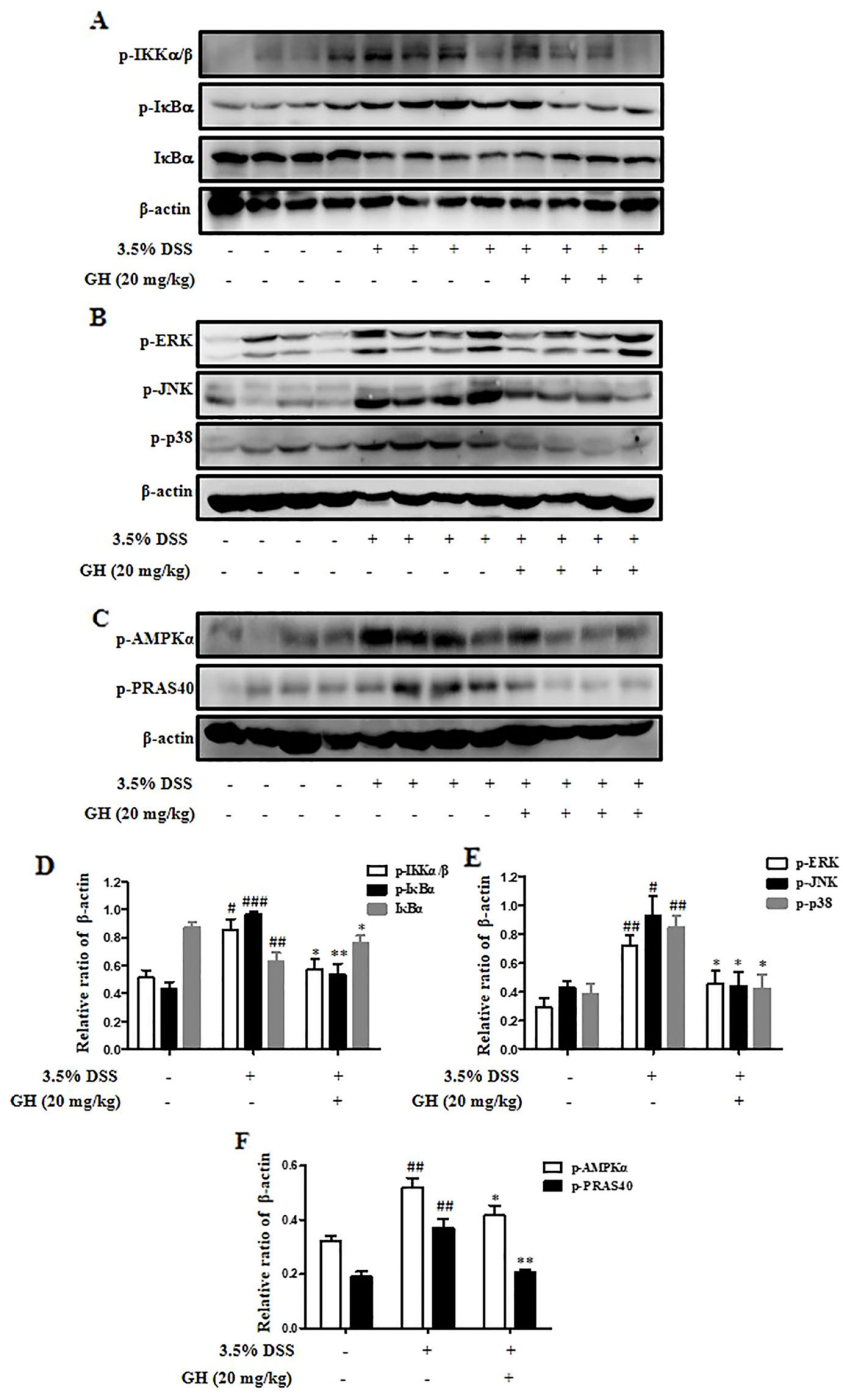
Gaudichaudione H (GH) inhibited nuclear factor-κB (NF-κB)/mitogen-activated protein kinase (MAPK) pathway and the phosphorylation of adenosine 5'-monophosphate-activated protein kinase-α (AMPKα) and proline-rich Akt substrate of 40 kDa (PRAS40) in colonic tissues in mice. The signaling molecules phospho-IκB kinase α/β (p-IKKα/β), p-IκBα, and IκBα in the nuclear factor-κB (NF-κB) pathway **(A)**, the signaling molecules phospho-extracellular signal-regulated kinase (p-ERK), phospho-c-Jun N-terminal kinase (p-JNK), and phospho-p38 (p-p38) in the MAPK pathway **(B)**, and protein p-AMPKα and p-PRAS40 **(C)** in the colons were analyzed using Western blot. **(D–F)** The density of proteins were calculated and normalized to β-actin. The data are the means ± SEM, n = 10. ^#^ *p* < 0.05, ^##^ *p* < 0.01, ^###^ *p* < 0.001 compared with control, *p < 0.05, **p < 0.01 compared with 3.5% DSS.

We detected the protein levels of p-IKKα/β, p-IκBα, IκBα, p-ERK, p-JNK, p-p38, p-AMPKα, and p-PRAS40 in colons. The results were consistent with the *in vitro* experiments. GH (20 mg/kg) inhibited NF-κB (**Figures 10A, D**) and MAPK (**Figures 10B, E**) pathways *in vivo*. In addition, GH could inhibit the phosphorylation of AMPKα and PRAS40 in the colon of mice (**Figures 10C, F**).

### Gaudichaudione H Alleviated Histopathological Damage and Infiltration of Macrophages in Colon of Dextran Sodium Sulfate-Treated Mice

**Figure 11:**
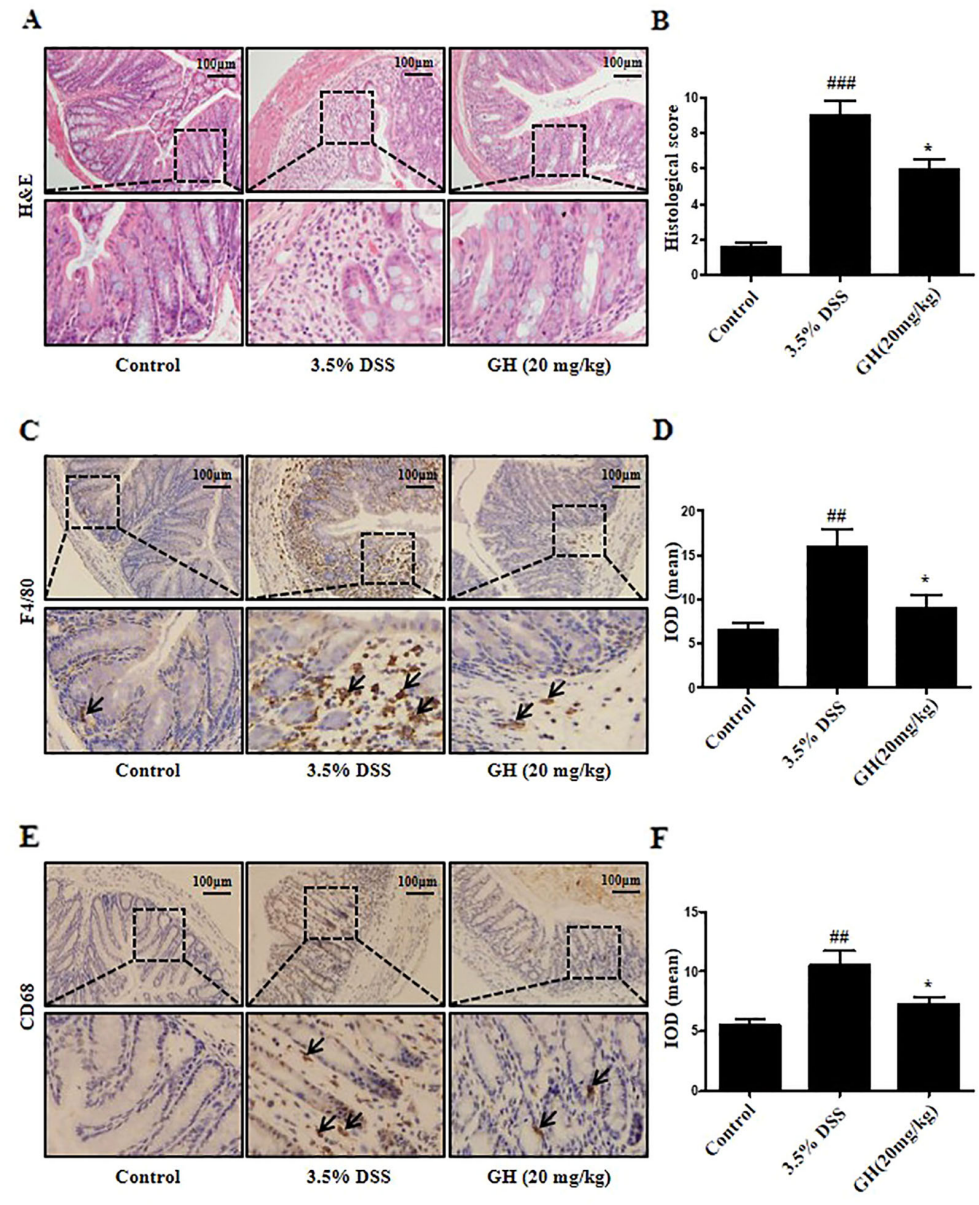
Gaudichaudione H (GH) alleviated histopathological damage and infiltration of macrophages in colon of dextran sodium sulfate (DSS)-induced mice. **(A)** Representative photomicrographs of sections from colonic samples stained with H&E (magnification ×10). **(B)** The effect of GH at doses of 20 mg kg^−1^ on microscopic scores of colonic injury in DSS-induced colitis model. **(C, E)** The immunohistochemistry (IHC) of F4/80 and CD68 in colon tissues was measured (magnification ×10). **(D, F)** The quantification of integral optical density (IOD) in the different groups using Image Pro-Plus 6.0. All data are the means ± SEM, n = 10. ^##^ *p* < 0.01, ^###^ *p* < 0.001 compared with control, *p < 0.05 compared with 3.5% DSS.

The 3.5% DSS group showed a diffuse destruction of intestinal wall and surface epithelium, crypt abscesses or loss, infiltrated lymphocytes and edema, while the control group showed a normal macroscopic appearance. GH treatment resulted in more intact surface epithelium and crypt glands than that of mice in the 3.5% DSS group (**Figure 11A**). GH attenuated the microscopic signs of colonic damage, as indicated by the histological score assessed by inflammation severity, the extent of injury, and crypt damage (**Figure 11B**). To characterize the infiltrated macrophage population in the colon, IHC was performed for macrophages phenotypic markers F4/80 and CD68. The results showed increased F4/80^+^ macrophages (**Figures 11C, D**) and CD68^+^ macrophages (**Figures 11E, F**) in colon of the 3.5% DSS group compared with the control group, and this effect was ameliorated by GH administration.

## Discussion

To our knowledge, there is no published report on the anti-inflammatory effect of GH. Therefore, in this study, we evaluated the anti-inflammatory activity of GH by measuring its effect on the production of NO in LPS-induced RAW 264.7 cells and attempted to explore its potential anti-inflammatory mechanism.

Bacterial LPS is a potent immune system activator, which can excessively activate macrophages triggering a series of signal activations including NF-κB and MAPK signaling pathways and the production of pro-inflammatory mediators including NO, reactive oxygen species (ROS), PGE2, IL-6, and TNF-α, further aggravating the progression of inflammation and leading to tissue damage. (Guha and Mackman, 2001; Biswas and Mantovani, 2010; Khan et al., 2017). Excessively activated macrophages play key roles in inflammation by generating various inflammatory mediators including pro-inflammatory cytokines, oxygen, and nitrogen species (Arango Duque and Descoteaux, 2014). NO is a typical signaling molecule in the pathogenesis of inflammation due to its overproduction from activated macrophages, which induces vasodilatation, tissue destruction, and the upregulation of leukocyte and endothelial adhesion molecules leading to enhanced inflammation (Sharma et al., 2007). Our data showed that GH inhibited NO production without affecting cell viability. To show how GH treatment reduced the level of NO in macrophages, we first detected the expression of iNOS and COX-2, two inflammatory mediators which are related to NO production. Protein expressions of iNOS and COX-2 were significantly reduced by GH in a dose-dependent manner.

Pro-inflammatory cytokines IL-6 and TNF-α are endogenous pyrogens that promote fever. In addition, IL-6 recruits monocytes to the inflammation site, promotes the maintenance of T helper 17 (Th17) cells, and inhibits T cell apoptosis and the development of regulatory T cells (Tregs). TNF-α induces vasodilation and loss of vascular permeability (Arango Duque and Descoteaux, 2014). We detected these two important pro-inflammatory cytokines, and our data showed that the protein level and mRNA expression of TNF-α and IL-6 were decreased by GH compared with the increased levels of these cytokines in LPS-induced RAW 264.7 cells.

As we all know, the activated NF-κB signaling pathway has a significant impact on the development of inflammation, and NF-κB, one of the major transcriptional factors, is a mediator of pro-inflammatory gene induction. The primary step for NF-κB activation is the inducible degradation of IκBα mediated by site-specific phosphorylation by an IKK complex (Fusella et al., 2017; Liu et al., 2017). As the MAPKs are also important in LPS-induced inflammation in macrophages, we investigated the effects of GH on the NF-κB and MAPK signaling pathways in macrophages. We found that GH inhibited the phosphorylation of the signal protein in these two pathways.

Since the transcription factors NF-κB and AP-1 are two major regulators in controlling iNOS, COX-2, IL-6, and TNF-α expression, we then observed the translocation of NF-κB and AP-1 in LPS-induced RAW 264.7 cells with or without GH treatments. Our data showed that GH obviously inhibited the translocation of p65, c-fos, and p-c-Jun from the cytoplasm to the nucleus.

The MyD88-dependent pathway is activated by the TLR4 ligand LPS. Upon ligand binding, TLRs recruit adaptor proteins MyD88, activating the IRAK1 and IRAK4, enabling the recruitment of TNF receptor-associated factor 6 (TRAF6), which phosphorylates transforming growth factor-β-activated kinase 1 binding protein 2/3 (TAB2/TAB3) and transforming growth factor-β-activated kinase 1 (TAK1), leading to the activation of the NF-κB and MAPK signaling pathway (Ajibade et al., 2013; Wang et al., 2017). Our data showed that GH downregulated the activation of TLR4-MyD88 signaling pathway in RAW 264.7 cells and BMDMs.

To further determine whether GH have similar anti-inflammatory effect on colonic inflammation *in vivo*, we used a mouse model of DSS-induced acute colitis. Our findings showed that GH led to an amelioration of symptoms of weight loss, blood in stool, diarrhea, and colon shortening, inflammatory mediators IL-6, TNF-α, iNOS, and COX-2 expression, the activation of NF-κB and MAPK pathways, AMPKα and PRAS40 phosphorylation, histopathological damage, and infiltration of macrophages in the colon of mice with DSS-induced colitis.

Accumulating evidence has demonstrated that small molecular compounds from Chinese herbal medicine are promising for effective anti-inflammatory therapeutics. There have been some reports on the anti-inflammatory activity of compounds in the genus *Garcinia* Linn. For example, oblongifolin C (OC) from *Garcinia yunnanensis* Hu. and nujiangexanthone A (NJXA) from *Garcinia nujiangensis* exerted an anti-allergic effect on immunoglobulin E (IgE)/Ag-induced mouse mast cells *in vitro*. In addition, nujiangexanthone A ameliorated the ovalbumin-induced asthma in a mouse model. Molecular mechanisms of OC and NJXA were shown to involve the inhibition of prostaglandin D2 (PGD2) generation, leukotriene C4 (LTC4) generation, and the degranulation reaction in IgE/Ag-stimulated mast cells (Lu et al., 2015; Lu et al., 2016). Cambogin isolated from *Garcinia esculenta* Y. H. Li. inhibited DSS-induced colitis in mice through the upregulation of Treg cell stability and function (Lu et al., 2018). In addition, other compounds such as 1,3,5,7-tetrahydroxy-8-isoprenylxanthone (TIE) from the twigs of *G. esculenta*, kolaviron from the seeds of *Garcinia kola*, and β-mangostin from *Garcinia mangostana* Linn. showed potential anti-inflammatory effects (Abarikwu, 2014; Syam et al., 2014; Zhang et al., 2015). However, more research is still necessary to explore the anti-inflammatory effects of GH, the genus *Garcinia* Linn., and Chinese medicine.

AMPKα, an energy regulator of cellular metabolism activated by glucose deprivation, heat shock, oxidative stress, and ischemia, upregulates mitochondrial biogenesis, glucose uptake, and lipid metabolism to produce energy and restore ATP levels and energy balance (Ye, 2013). Some reports found that the inhibition of p-AMPKα played role in ameliorating inflammation which was similar to our results. For example, elevated phosphorylation of AMPK was reported to promote NF-κB activation and inflammation in LPS-induced THP-1 cells (Wang et al., 2015). The results may reveal that macrophages activated by LPS need more energy and have an increasing cellular metabolism, and GH inhibits the activation of AMPKα to reduce the energy supplementation and inhibit subsequent signaling activation. PRAS40 is a component of the mammalian target of rapamycin complex 1 (mTORC1), which is an endogenous inhibitor of mTORC1 (Jhanwar-Uniyal et al., 2017). One research reported that PRAS40 could elevate NF-κB transcriptional activity through association with p65 (Zhu et al., 2017). Hence, it was showed that activation of PRAS40 could enhance inflammatory response, while our data showed that GH inhibited the activation of PRAS40.

However, to fully understand the anti-inflammatory effects and mechanisms of GH, further study needs to be performed, such as the research on the effects of GH on other inflammatory signaling pathways, specific target molecules, and other inflammatory diseases such as sepsis, passive systemic allergic reactions, asthma, etc.

In summary, our results indicated for the first time that GH could inhibit inflammation in macrophages and DSS-induced colitis in mice. GH inhibited pro-inflammatory mediators and cytokines including NO, iNOS, COX-2, TNF-α, and IL-6 in LPS-stimulated RAW 264.7 cells by blocking the activation of TLR4-mediated NF-κB and MAPK signaling pathways and nuclear translocation of transcription factors NF-κB and AP-1. There was a similar effect of GH in BMDMs. GH could also inhibited the phosphorylation of AMPKα and PRAS40 in BMDMs. Moreover, GH alleviated DSS-induced colitis in mice by alleviating weight loss, stool consistency change, hematochezia, colon shortening, IL-6, TNF-α, iNOS, and COX-2 expression, activation of NF-κB and MAPK pathways, and the phosphorylation of AMPKα and PRAS40 in colon tissues, as well as colonic damage and infiltration of macrophages in colon of mice with DSS-induced colitis. These findings suggested that GH could be potentially developed as a potential anti-inflammatory agent for clinical and medical applications, and GH may be a new therapeutic compound for the inhibition of macrophage-mediated inflammatory diseases, such as IBD, sepsis, and rheumatic arthritis.

## Data Availability Statement

All datasets generated for this study are included in the article/**Supplementary Material**.

## Ethics Statement

The animal study was reviewed and approved by the animal ethics committee of Shanghai University of Traditional Chinese Medicine.

## Author Contributions

YL and HX conceived and designed the experiments and revised the manuscript. YJ and LX performed the experiments and wrote the manuscript. PL, NK, and HT analyzed the data. WF, YT, and CZ collected the compound. All authors read and approved the final manuscript.

## Funding

This work was financially sponsored by grants from the National Natural Science Foundation of China (NSFC) Grants 81803545 and 81602990; Professor of Special Appointment (Eastern Scholar) at Shanghai Institutions of Higher Learning; the Three-year development plan project for Traditional Chinese Medicine (ZY(2018-2020)-CCCX-2001-02) for financial support.

## Conflict of Interest

The authors declare that the research was conducted in the absence of any commercial or financial relationships that could be construed as a potential conflict of interest.

The reviewer WD declared a shared affiliation with the authors, YJ, LX, WF, YT, PL, NK, CZ, HT, YL, HX, to the handling editor at time of review.
