# Supplementary material for: Gaudichaudione H Inhibits Inflammatory Responses in Macrophages and Dextran Sodium Sulfate-Induced Colitis in Mice
Source: Front Pharmacol. 2020 Jan 17;10:1561. doi: 10.3389/fphar.2019.01561 (PMC6978770; doi:10.3389/fphar.2019.01561)
Supplement: Supplementary file 1 [file DataSheet_1.docx]

Supplementary Material

## Supplementary materials and methods

UPLC chromatogram

^1^H-NMR and ^13^C-NMR spectra were measured on a Bruker AV-400 spectrometer and calibrated by the solvent peak used (pyridine-d6). Ultra performance liquid chromatography (UPLC) was performed using a Waters Acquity. UPLC I class system (Waters, Milford, MA, USA), equipped with a binary solvent delivery system, an auto sampler and a photodiode array detection system. Chromatography was performed on a Waters ACQUITY BEHC18 column (2.1 mm × 100 mm I.D., 1.7 μm, Waters). The mobile phase consisted of (A) 0.1% formic acid in water and (B) acetonitrile. The UPLC eluting conditions were as follows: 60-70% B (0-2 min), 70-90% B (2-7 min), 90-100% B (7-12 min) and 100%-100% B (12-13 min). The flow rate was maintained at 0.3 mL min ^-1^.The column and auto sampler were maintained at 40 and 10°C respectively. The detection wavelengths were set at 238, 254 and 360 nm.

## Supplementary Figure


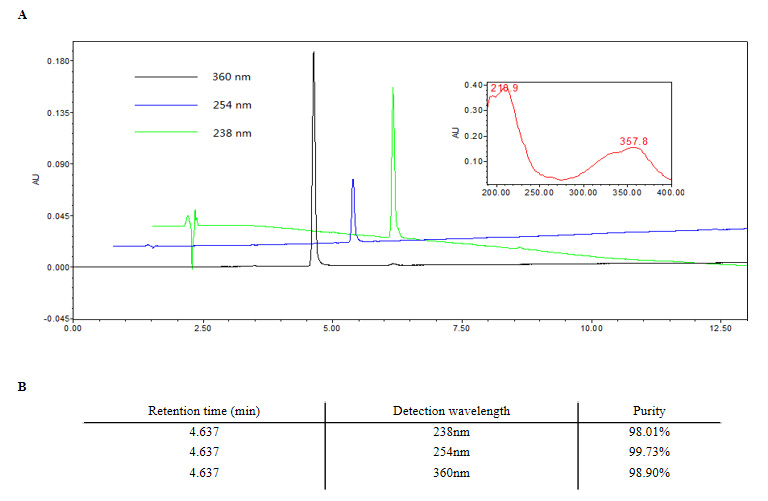


**Supplementary Figure 1.** **(A)** UPLC chromatogram of Gaudichaudione H. Column: Waters ACQUITY UPLC®BEH C18 (1.7 μm, 2.1 * 100 mm); mobile phase: (C) acetonitrile and (D) 0.1% formic acid in water, in gradient mode as follows: 60-70% B (0-2 min), 70-90% B (2-7 min) , 90-100% B (7-12 min) and 100%-100% B (12-13 min); detection wavelength: 238, 254 and 360 nm; and flow rate: 0.3 mL min ^-1^. **(B)** Purity detection of Gaudichaudione H.


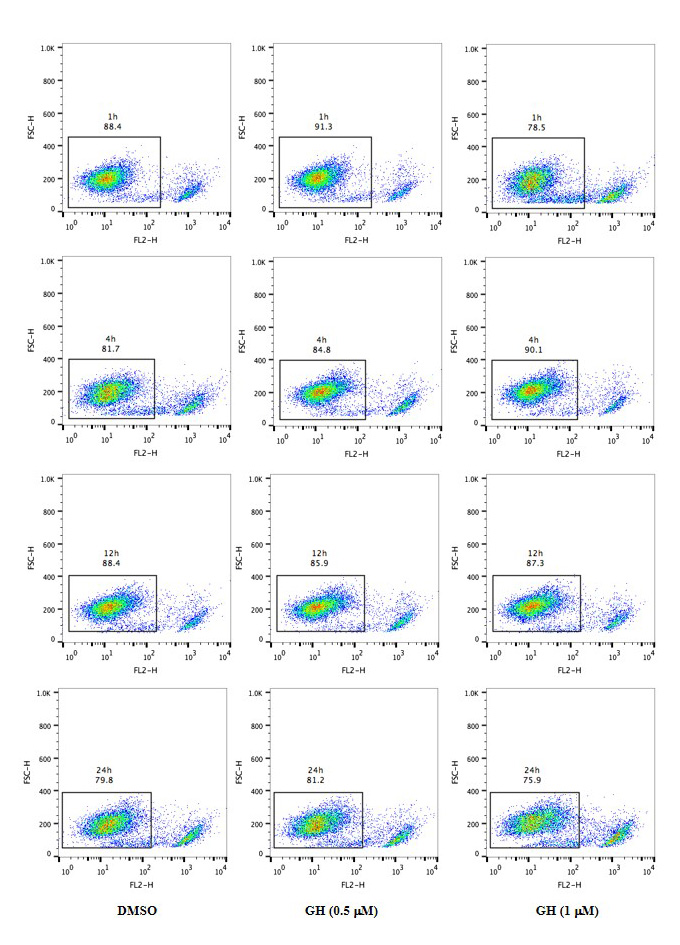


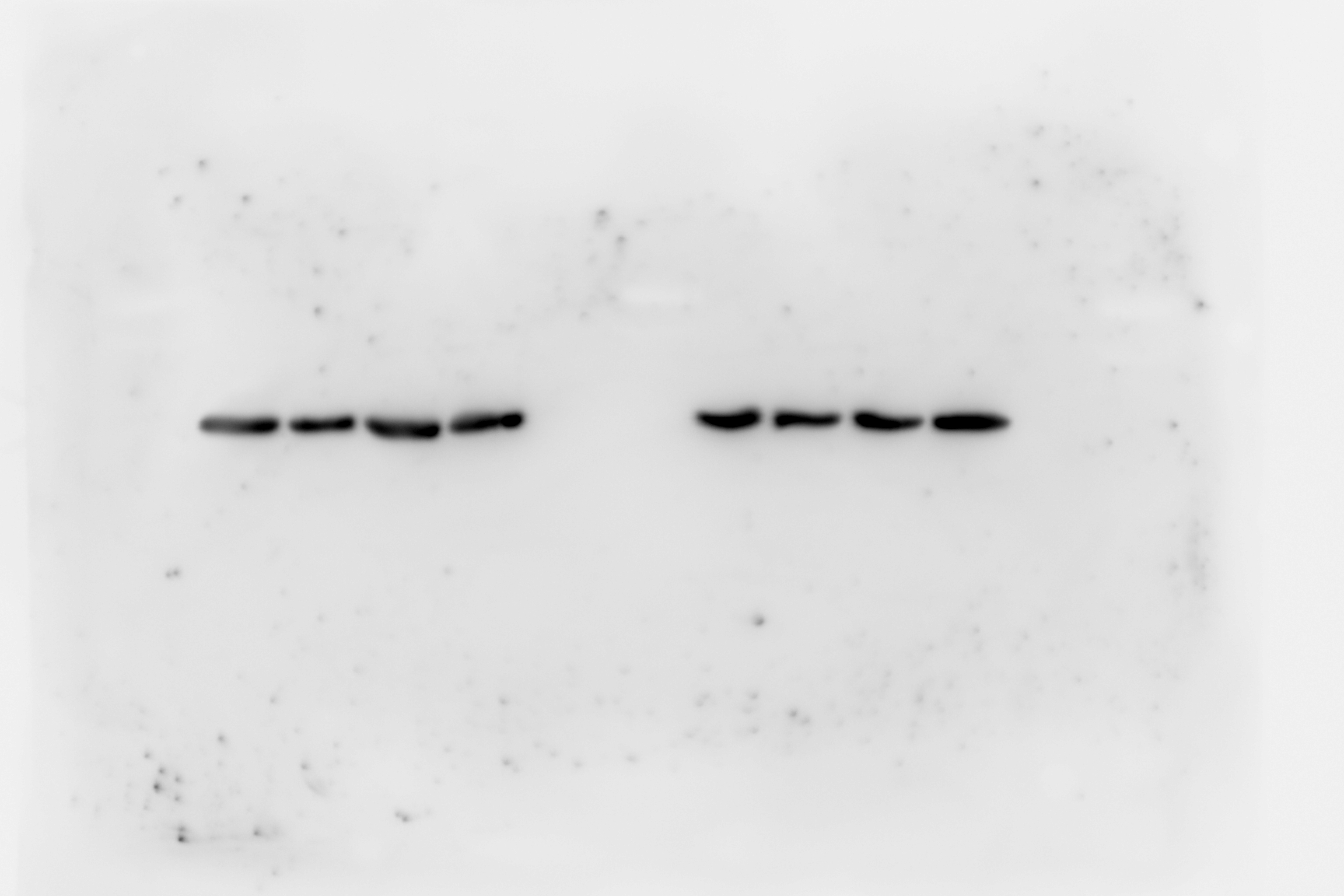
**Supplementary Figure 2.** GH did not affect the cell viability of Raw 264.7 cells at different time points. PI/AnnexinV analysis was performed to evaluate the effect of GH on the cell state at different time points (1h, 4h, 12h, 24h).
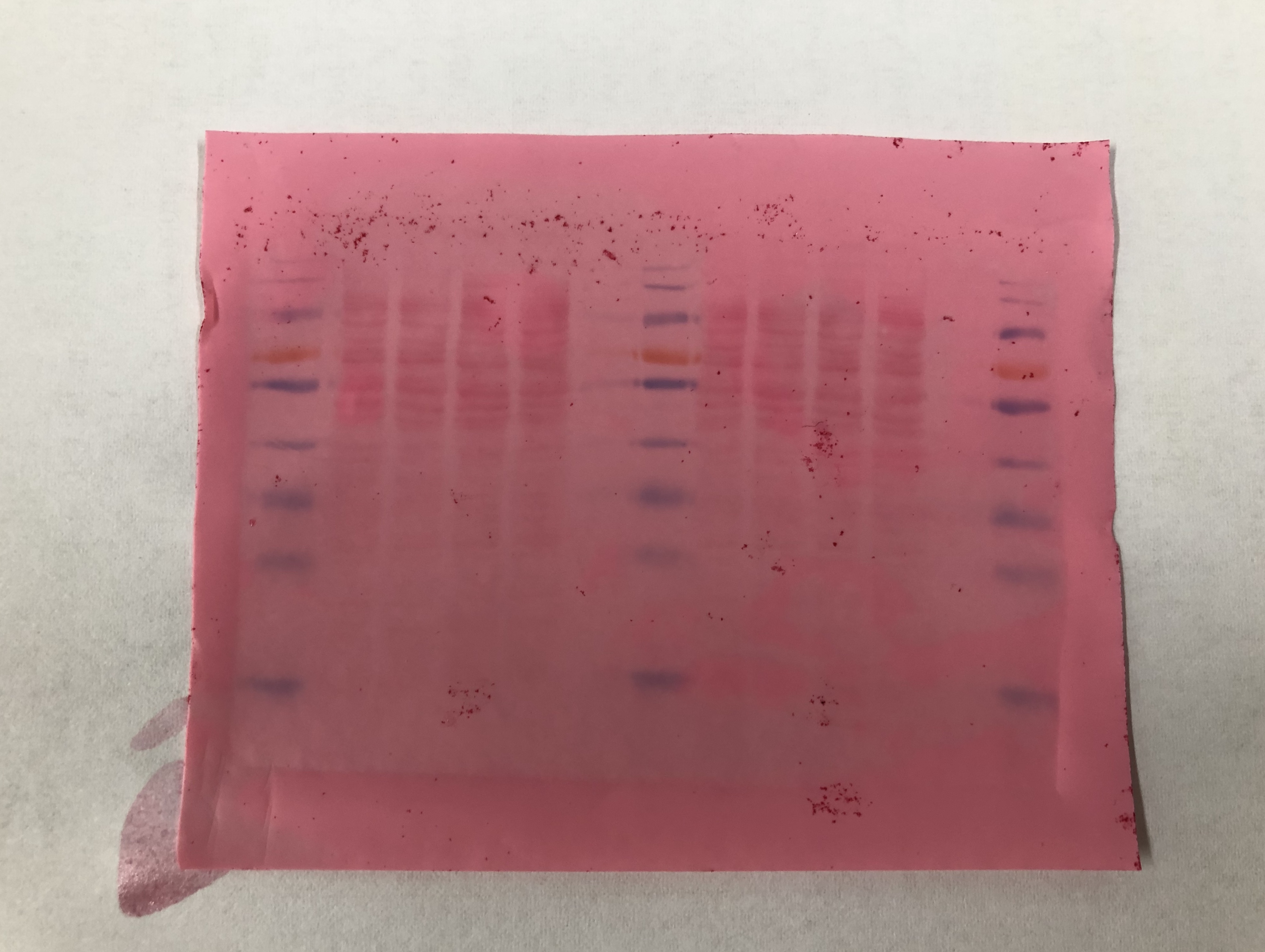


**Supplementary Figure 3.** The ponceau staining as loading control and the actin for the western blots.


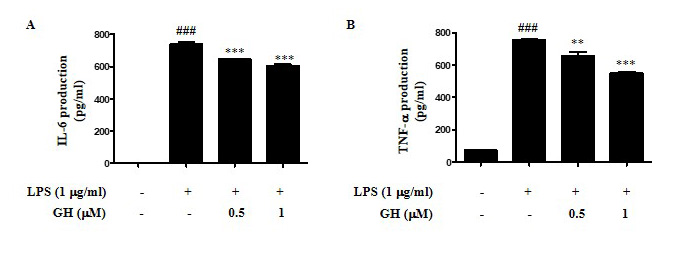


**Supplementary Figure 4.** GH inhibited cytokine IL-6 and TNF-α production and rescued LPS-induced inflammation in RAW 264.7 cells. RAW 264.7 cells were cultured at 5×10^5^ cells well^-1^ in 12-well plates, and LPS (1 μg mL^-1^) was pretreated for 1 h and then co-treated with GH for 24h. IL-6 (A) and TNF-α (B) in the cell supernatants were assayed using ELISA. ###*p*<0.001 compared with control, ***p*<0.01, ****p*<0.001 compared with LPS.


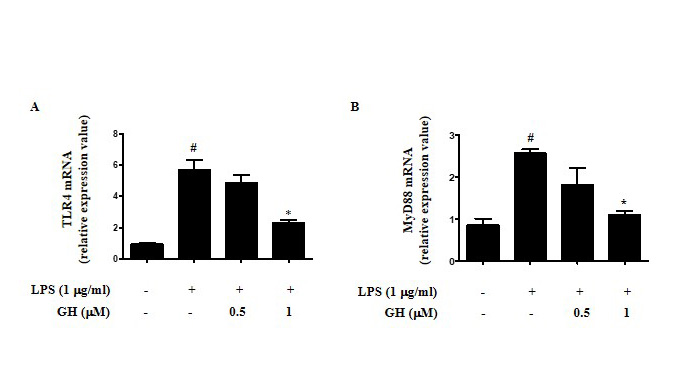


**Supplementary Figure 5.** GH inhibited TLR4 and MyD88 mRNA expression in LPS-stimulated RAW 264.7 cells. RAW 264.7 cells were cultured at 5×10^5^ cells well^-1^ in 12-well plates, and GH was pretreated with GH for 1 h and then co-treated with LPS (1 μg mL^-1^) for 15 minutes.


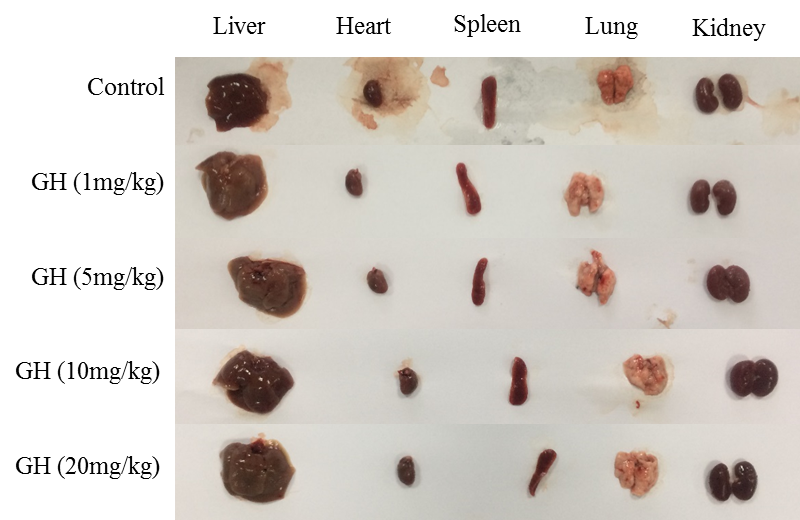


**Supplementary Figure 6.** The liver, heart, spleen, lung and kidney tissues of the control group and the GH group were observed. GH was administered once a day for 10 days. The mice were sacrificed at Day 10 and the liver, heart, spleen, lung and kidney tissues were taken.
